# Multi-objective route optimization for electric vehicle hazardous materials transportation in uncertain environments

**DOI:** 10.1038/s41598-025-32134-3

**Published:** 2025-12-22

**Authors:** Qian Zhang, Zejian Zhang, Chao Ma

**Affiliations:** 1 School of Automobile and Traffic Engineering, Hubei University of Arts and Science, Xiangyang, 441053 China; 2 Hubei Institute of Logistics Technology, Xiangyang, 441002 China; 3 Hubei Key Laboratory of Power System Design and Test for Electrical Vehicle, Hubei University of Arts and Science, Xiangyang, 441053 China

**Keywords:** Engineering, Mathematics and computing

## Abstract

This paper focuses on the application of electric vehicles in the transportation of Category 9 hazardous materials. Given the high requirements for safety and timeliness in hazardous materials transportation, this study first comprehensively considers the impacts of population density uncertainty and cargo volume changes on transportation risks and power consumption. Furthermore, a multi-objective path optimization model is developed. The model aims to minimize transportation risks, reduce costs, and maximize customer satisfaction. It includes constraints on accident probability, cargo volume, and time windows. To solve this model, an improved Non-dominated Sorting Genetic Algorithm II (NSGA-II), named H-NSGA-II, is designed. It is based on the fusion characteristics of the greedy algorithm and the traditional NSGA-II algorithm. Through case validation, it is found that the algorithm can efficiently obtain high-quality Pareto solutions. Compared with the original NSGA-II algorithm, the optimal transportation risk, transportation cost, and average customer satisfaction are improved by 14.40%, 12.81%, and 13.53%, respectively. The research results can provide decision-making support for the safe, economical, and green distribution of urban Category 9 hazardous materials.

## Introduction

With the rapid development of China’s chemical industry, the demand and transportation volume of hazardous materials have grown significantly. Since the 21st century, the hazardous materials vehicle routing problem (VRP) has become a hotspot in the field of path optimization. Due to the characteristics of inflammability, explosiveness, radioactivity, and corrosiveness of such materials, any accident involving them may cause significant impacts. These impacts include not only economic losses to enterprises and threats to the personal safety of surrounding personnel, but also environmental pollution. Against this backdrop, the rational arrangement of vehicle routes is of vital importance to the safety of hazardous materials transportation (HMT). Notably, the Ministry of Transport of China has indicated that electric vehicles (EVs) can be used for transporting Category 9 hazardous materials. This provides a solid foundation for the implementation of EVs in HMT scenarios.

In fact, the technical characteristics of EVs provide a feasibility guarantee for their application in HMT. In recent years, with social development and increasing environmental pollution, the concept of low carbon has become a key focus of social development. Some data show that the exhaust of fuel vehicles is one of the main sources of greenhouse gas emissions^1^. Wang et al.^2^ studied the impact of promoting EVs on China’s carbon emissions. They found that replacing fuel vehicles with EVs is crucial. This replacement significantly advances the automotive industry’s development strategy and adjusts energy structure policies. Alanazi^3^ conducted an in-depth study on the gradual adoption of EVs, demonstrating their effectiveness in reducing carbon emissions and mitigating air pollution. This confirms that replacing conventional fuel-powered vehicles with EVs benefits environmental protection. Regarding operational costs, Liu et al.^4^ compared battery EVs with internal combustion engine vehicles based on total costs. Their research found that battery EVs with a driving range below 200 miles incur lower costs than internal combustion engine vehicles when used for over eight years. Zhang et al.^5^ performed a comparative analysis of EVs and hydrogen fuel cell vehicles across power sources, fuel storage and transportation, infrastructure development, and vehicle costs. The study revealed that electric passenger cars outperform hydrogen fuel cell vehicles in terms of economic efficiency, safety, and environmental impact. These advantages have facilitated the widespread application of EVs in urban distribution and logistics, laying a technical and practical foundation for their expansion into HMT scenarios.

Despite the promising prospects of EVs in hazardous material transport, significant gaps remain in current route optimization research. On one hand, in the hazardous materials VRP domain, many scholars prioritize deterministic factors such as transport risks and costs. For instance, Jiang et al.^6^ proposed a risk assessment model for HMT considering vehicle types and waiting times. A three-objective vehicle routing model based on total transportation cost, transportation risk and average vehicle redundancy was established. A hybrid multi-objective evolutionary algorithm based on variable neighborhood search was designed to solve the problem. Eren and Tuzkaya^7^ addressed the dual-objective problem of medical waste transport during COVID-19, balancing safety and route distance. Li et al.^8^ studied the transport of dangerous goods in the context of terrorist attacks. In order to solve this problem, a systematic risk management method based on game theory is proposed. Finally, they took the actual road network in Beijing as an example for path optimization. While deterministic factors are critical, uncertainties (e.g., accident consequences and parameters) are equally important. Examples include, Zhao et al.^9^ studied the problem of optimizing transportation routes for medical hazardous materials under demand uncertainty. Taking the current situation of the COVID-19 epidemic in Wuhan, China as a research case, they demonstrated the feasibility of the model. Zhang et al.^10^ established an uncertain model based on the uncertain parameters of customer demand. Then they transformed the model into a robust model with adjustable parameters. Finally, they improved the reinforcement learning algorithm based on deep Q-learning to solve the robust model. Holeczek^11^ compared risk models, highlighting the impact of dynamic vehicle load changes. Zhang et al.^12^ considered the actual load-related risks in the HMT for the multi-depot heterogeneous VRP with time windows. They established a multi-objective optimization model and solved it through a hybrid multi-objective evolutionary algorithm and a two-stage algorithm. Han and Zhu^13^ evaluated transportation risks by the number of population exposures on past road segments. They used a travel speed-based time-dependent function to capture the dynamic characteristics of urban road networks. Then, an optimization model was constructed with the objectives of minimizing total transportation costs and transportation risks. Finally, a multi-objective evolutionary algorithm was designed based on Non-dominated Sorting Genetic Algorithm II (NSGA-II) combined with a simulated annealing algorithm. Wang and Liang^14^ explored the driving rules of HMT vehicles. They mainly considered the travel rules of vehicles and personnel on the road, as well as the distribution of population and environmentally sensitive areas along the route. Subsequently, a bi-objective model for HMT under time-varying conditions was proposed, and a comprehensive model-solving method integrating multiple algorithms was designed. Zahiri^15^ tackled multi-period stochastic planning for uncertain hazardous transport networks. Notably, most studies overlook customer satisfaction in HMT.

On the other hand, in the electric VRP research field, the multi-objective collaborative optimization capability is limited. Eslamipoor^16^ proposed a VRP model for picking up and delivering goods to different warehouses. They considered the impacts of vehicle capacity and energy consumption varying with load. The model aims to reduce both customer costs and waiting time simultaneously. Deng et al.^17^ constructed a multi-objective optimization model based on the battery swapping mode. They considered EV distribution route planning, charging management, and customer satisfaction in the model. Finally, the non-dominated sorting genetic algorithm was used to solve the proposed mathematical model. Zhao et al.^18^ designed a model for the electric VRP under time-varying traffic conditions to reduce the total logistics distribution cost. The model takes into account factors such as time-varying road network traffic, road types, customers’ time window requirements, the freshness of fresh products, and queuing for charging during the journey. They designed an improved adaptive ant colony algorithm to solve the model. Qiang et al.^19^ constructed an EV routing model with time windows to minimize the total cost. They also proposed a hybrid adaptive genetic algorithm to solve the model. Cai et al.^20^ established a multi-objective optimization model with the objectives of minimizing distribution costs and maximizing customer satisfaction, considering electricity consumption. They also designed a multi-objective genetic algorithm with fast non-dominated sorting to solve the model. In addition, studies have found that the energy consumption of EVs varies under different driving speeds and vehicle loads. Xie et al.^21^ studied the influence of different parameters on the performance characteristics of EVs. The results showed that the main factors affecting energy consumption and driving range are the average speed, operation time, and frequency distribution of braking processes. Wu and Tian^22^ investigated the impact of dynamic loads on the energy consumption rate of EVs. A mathematical optimization model was constructed by considering traditional factors such as battery loss, charging station service time, and time-of-use fees. They also used an improved genetic algorithm to solve the model. Cheng et al.^23^ studied the spatio-temporal distribution prediction of EV charging loads. They constructed an energy consumption model for EVs and a charging load prediction model. The models took into account temperature, traffic conditions, and the subjective of EV owners in different scenarios. Lee et al.^24^ proposed an energy-saving speed planning strategy based on reinforcement learning to reduce vehicle energy consumption under various driving conditions. They also developed a model-based reinforcement learning algorithm to solve the model. Miri et al.^25^ established an energy consumption model for EVs to accurately estimate their driving range. They also developed a driver model to control vehicle speed and represent human driving behavior. At present, most existing models use traditional fuel-powered vehicles for transporting hazardous chemicals. Research on route optimization for transporting Class 9 hazardous materials using EVs is almost non-existent. Moreover, most studies do not consider the driving range, charging needs, etc., of EVs.

In real transportation scenarios, when EVs are used for the transportation of Class 9 dangerous goods, route planning is affected by multiple factors. These include changes in population density, fluctuations in cargo volume, transportation risks, power consumption, and customer satisfaction. However, existing research has not proposed a multi-objective route optimization scheme for such transportation scenarios that comprehensively considers the above factors. Overall, this study presents significant differences from previous research. In the field of hazardous materials VRP, this paper pioneers the use of EVs for HMT, distinguishing itself from references^6–15^. Unlike prior studies^6–8^, it examines the influence of uncertain variables-population density, load fluctuations-on transportation risks. In contrast to literature^9–15^, this research integrates three objectives: transportation risk, cost, and customer satisfaction, while analyzing how uncertainties impact transportation risks. Within the electric VRP domain, this study formulates an electricity consumption model with time window constraints, a departure from references^16,17^. Differing from^18,19^, it not only accounts for customer satisfaction but also develops a load-sensitive electricity consumption model. Compared with^22,23,25^, it broadens the research scope by incorporating both customer satisfaction and time window constraints. In short, this study has achieved breakthroughs in multiple aspects, and the specific contributions are shown in Table 1.

**Table 1:** Comparison of studies.

Author	Vehicle type	objective functions	Load change effect	Factors affecting the transportation of hazardous materials	Influence of population density	Time window constraints	Influence of population density		
	Electric vehicle	Fuel vehicles		With	Deterministic Factors	Uncertainty factors	With	With	With
Jiang et al.^6^		√	Transportation Cost, Transportation Risk, Travel Time		√			√	
Eren et al.^7^		√	Transportation Safety, Transport distance		√			√	
Li et al.^8^		√	Transportation Risk		√				
Zhao et al.^9^		√	Total cost, Transportation Risk			√			
Zhang et al.^10^		√	Transportation Cost	√		√			
Holeczek et al.^11^		√	Transportation Cost, Transportation Risk	√		√			
Zhang et al.^12^		√	Total cost, Transportation Risk			√			
Han et al.^13^		√	Total cost			√	√		
Wang et al.^14^		√	Transportation Cost, Transportation Risk, Travel Time			√	√		
Zahiri et al.^15^		√	Total cost, Transportation Risk			√			
Eslamipoor et al.^16^	√		Total cost, Carbon emissions	√					
Deng et al.^17^	√		Total cost, Customer satisfaction, Battery loss					√	
Zhao et al.^18^	√		Total cost, Product Freshness					√	
Qiang et al.^19^	√		Total cost, Carbon emissions						
Cai et al.^20^	√		Total cost, Customer satisfaction	√				√	√
Wu et al.^22^	√		Total cost, Battery loss	√					
Cheng et al.^23^	√		None						√
Miri et al.^25^	√		None						√
This paper	√		Total cost, Transportation Risk, Customer satisfaction	√		√	√	√	√

To fill the research gaps, this paper studies the transportation of Class 9 dangerous goods by EVs. The goal is to build a route planning system. It considers multi-source uncertainties and multi-objective optimization. Specifically, we quantify how population density and cargo volume affect transportation risks and power consumption. Then, we create a three-objective model. It aims to minimize transportation risks, cut costs, and maximize customer satisfaction. We combine the greedy algorithm with NSGA-II. Also, we design three types of neighborhood operators. They help solve the model efficiently in complex situations. Our study has three main innovations. First, we consider population density and load changes in risk assessment. Second, we propose a framework for multi-objective optimization. It balances safety, economy, and service goals. Third, case studies show that our improved algorithm’s Pareto solutions are better. Compared with traditional methods, the optimal transportation risk drops by over 14.40%, costs by 12.81%, and average customer satisfaction rises by 13.53%. This research provides useful tools for government regulation and business operations.

The remaining structure of this paper is as follows. Section Problem description describes research assumptions and symbols. Section Model construction constructs the relevant model. Section Design of multi-objective genetic algorithm designs the solution algorithm. Section Analysis of case simulation results presents simulation results. Section Conclusion concludes the full text.

### Problem description

The multi-objective HMT problem for EVs under uncertain transportation risks can be described as follows. Within an HMT network, there is a dedicated hazardous materials distribution center equipped with sufficient inventory and a fleet of EVs. A team of EVs is dispatched from the center to provide logistics and distribution services for specified customers within pre-defined time windows. Consistent with Cai et al.^20^, a delivery is deemed invalid if an EV arrives more than 2 hours beyond the customer’s latest acceptable time window. Under the constraint of not exceeding the vehicle’s maximum cargo capacity, a single EV can serve multiple customers, with each customer’s demand being indivisible. Every vehicle must return to the depot upon completing all assigned deliveries. Due to battery capacity limitations, if an EV’s remaining power is insufficient to support subsequent delivery tasks or return to the depot, the vehicle must seek a suitable charging station for power replenishment. To enhance operational flexibility, partial charging is permitted: EVs can recharge any amount of electricity as needed, up to the battery’s maximum capacity. A multi-objective EV routing model for hazardous materials is thus established, with the core optimization objectives of minimizing transportation risk, minimizing total transportation costs, and maximizing average customer satisfaction—all aimed at optimizing hazardous materials distribution routes under uncertainty.

This paper models a mixed-integer linear programming model on the complete directed graph $M$. Among them, customers are modeled as vertices. The paths between logistics nodes are modeled as arcs of the directed graph. Specifically, it is expressed as $M = (V_{0,N + 1} \cup F,A)$. Among them, $V_{0,N + 1}$ is the vertex set ($V_{0,N + 1} = V \cup \{ o\} \cup \{ N + 1\}$), where vertices 0 and $N + 1$ represent the distribution centers. $V$ represents the set of customers ($V = \{ 1,2,...,N\}$). $F$ represents the set of charging stations ($F = \{ 1,2,...,n\}$). $K$ represents the set of vehicles ($K = \{ 1,2,...,k\}$). $A$ represents the set of graph arcs ($A = \{ (i,j)|i \in V_{0,N + 1} \cup F,j \in V_{0,N + 1} \cup F,i \ne j\}$). The definitions of relevant parameters and variables in the model are shown in Table 2. The model is established based on the following assumptions:

**Table 2:** Parameter and variable definitions.

Symbol type	Symbol	Meaning
Set	$V_{0,N + 1}$	Vertex set,$V_{0,N + 1} = V \cup \{ o\} \cup \{ N + 1\}$
	$V$	Customer set,$V = \{ 1,2,...,N\}$
	$F$	Charging station cluster,$F = \{ 1,2,...,n\}$
	$K$	Vehicle Assembly,$K = \{ 1,2,...,k\}$
	$A$	Graph Arc Set,$A = \{ (i,j)|i \in V_{0,N + 1} \cup F,j \in V_{0,N + 1} \cup F,i \ne j\}$
Parameter	$R$	Hazard area radius of impact
	$S_{ij}$	Area of the hazard area
	$d_{ij}$	Distance between demand points $(i,j)$
	$N_{ij}$	The number of people at risk
	$P_{ij}$	Probability of dangerous accidents
	$w_{ij}$	The vehicle load from node $i$ to node $j$
	$R_{th}$	Risk threshold
	$P_{th}$	Accident probability threshold
	$W$	Vehicle capacity
	$h_{i}$	The demand of node $i$
	$t_{i}^{k}$	The time when vehicle k arrives at customer node $i$, $i \in V$,$k \in K$
	$t_{i,wait}^{k}$	The possible waiting time for the vehicle at node $i$,$i \in V$
	$C_{veh}$	Unit vehicle use cost (YUAN)
	$C_{pelec}$	Average unit charging cost (YUAN/kW•h)
	$T_{i}^{1}$	Earliest time window for customer $i$,$i \in V$
	$T_{i}^{2}$	Latest time window for customer $i$,$i \in V$
	$b_{ij}$	Unit distance power consumption coefficient (kW•h/km)
	$C_{wait}$	Waiting cost per unit time (YUAN/hour)
	$C_{penal}$	Punishment cost per unit time (YUAN/hour)
	$C_{buy}$	Vehicle purchase cost (YUAN)
	$B$	Electric vehicle power battery capacity (kW•h)
	$B_{j}^{k}$	Vehicle k’s battery level when leaving node $j$,$j \in V$
Decision variable	$x_{ij}^{k}$	$x_{ij}^{k} = 1$ indicates that vehicle k is in use, otherwise $x_{ij}^{k} = 0,k \in K,j \in V \cup F$
	$y_{ij}^{k}$	$y_{ij}^{k} = 1$ Indicates that vehicle $k$ goes to charging station $j$ for charging after visiting node $i$, otherwise $y_{ij}^{k} = 0,k \in K,i \in V_{0} ,N + 1,j \in F$
	$u_{j}^{k}$	Represents the amount of electricity that vehicle k replenishes at charging station $j$
Intermediate variable	$r_{ij}$	The risk of vehicle k from node $i$ to node $j$
	$R_{total}$	Total Transportation Risk
	$C_{fixed}$	Vehicle fixed costs
	$C_{ch\arg e}$	Charging cost
	$C_{time}$	Time window penalty cost
	$\rho$	Population density

1. All EVs have the same performance.
2. All EVs are driving at a constant speed on a flat road, and the speed is the same.
3. EVs mainly transport Category 9 hazardous materials such as lithium battery packs, capacitors, and lithium battery energy storage systems, which can have adverse effects when subjected to severe collisions.
4. All vehicles start with a full charge, and they only leave the charging pile after being fully charged.
5. Without considering traffic impacts such as congestion, it is deemed invalid if the vehicle arrives 2 hours later than the customer’s latest time window.

### Model construction

To formulate a reasonable route plan, this section takes steps as follows. First, it analyzes the relevant hazardous chemical risk impacts. Then, it considers distribution costs. Finally, it introduces the satisfaction problem. In this way, a route distribution model meeting the optimization plan is established.

### Dynamic risk model analysis

#### Population density analysis

Population density is an important factor in transportation risk assessment. The population density in traditional risk models is generally given a certain value based on experience. Considering that the uncertainty of population density will affect the accuracy of risk assessment, interval numbers are used to represent population density. That is, population density is expressed as $\rho = \left[ {\rho^{ - } ,\rho^{ + } } \right]$.

#### Risk model analysis

**Fig. 1:**
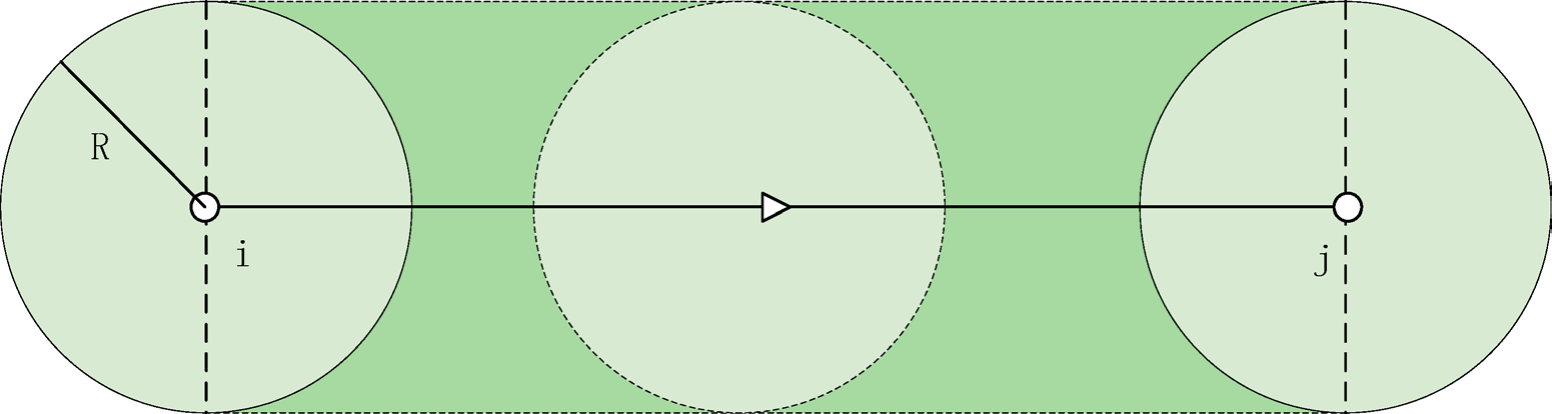
Schematic diagram of impact range area.

The traditional risk model defines the product of the accident probability and the number of affected people as the transportation risk value. In the HMT network, assuming the radius of the affected area in case of danger is R, then people within the radius R may be potentially affected by the danger. Therefore, the area of the affected range is defined as shown in Fig 1, and its area formula is:

1 $$S_{ij} = 2R \times d_{ij} + \pi \times R^{2} ,\forall (i,j) \in A,$$

Since the product of population density and the area affected by danger is the number of people affected by danger, it is expressed as:

2 $$N_{ij} = S_{ij} \times \rho ,\forall (i,j) \in A,$$

Let P is the coefficient of the accident probability and the transportation distance, then the accident probability can be expressed as:

3 $$P_{ij} = P \times d_{ij} ,\forall (i,j) \in A,$$

In addition to the accident probability and the number of affected people, the dynamic change of the cargo volume is also a key factor affecting the risk assessment. Different cargo volumes of vehicles will lead to different consequences in case of an accident at the same location. This paper refers to literature^26^ and assumes that the risk impact consequence has a linear relationship with the cargo volume, that is:

4 $$r_{ij} = P_{ij} \times N_{ij} \times \frac{{w_{ij} }}{W},\forall (i,j) \in A,$$

Since the cargo demand hi of customer node $i$ is an uncertain variable, the dynamic cargo volume of vehicle k on the delivery path $\left( {j - 1,j} \right)$ is:

5 $$w_{{L_{j - 1}^{k} L_{j}^{k} }} = \sum\limits_{L = m}^{{n_{k} }} {h_{{L_{j}^{k} }} } ,j \in V,k \in K,$$

The risk of vehicle k passing through section m is:

6 $$r_{{L_{j - 1}^{k} L_{j}^{k} }} = \sum\limits_{L = m}^{{n_{k} }} {\frac{{h_{{L_{j}^{k} }} }}{W}} \times P_{{L_{j - 1}^{k} L_{j}^{k} }} \times N_{{L_{j - 1}^{k} L_{j}^{k} }} ,j \in V,k \in K,$$

The cumulative risk of the delivery path of vehicle k is:

7 $$R_{k} = \sum\limits_{m = 1}^{{n_{k} }} {\sum\limits_{L = m}^{{n_{k} }} {\frac{{h_{{L_{j}^{k} }} }}{W}} \times P_{{L_{j - 1}^{k} L_{j}^{k} }} \times N_{{L_{j - 1}^{k} L_{j}^{k} }} } ,j \in V,k \in K,$$

The total transportation risk is:

8 $$R_{total} = \sum\limits_{k = 1}^{e} {\sum\limits_{m = 1}^{{n_{k} }} {\sum\limits_{L = m}^{{n_{k} }} {\frac{{h_{{L_{j}^{k} }} }}{W}} \times P_{{L_{j - 1}^{k} L_{j}^{k} }} \times } } d_{{L_{j - 1}^{k} L_{j}^{k} }} \times \left( {2R \times d_{{L_{j - 1}^{k} L_{j}^{k} }} + \pi \times R^{2} } \right) \times \left[ {\rho^{ - } ,\rho^{ + } } \right],j \in V,k \in K,$$

### Deterministic transformation of the uncertain risk model

To solve the model with interval-valued population density, it is necessary to transform the uncertain risk formulation into a deterministic equivalent. This transformation allows the application of conventional optimization algorithms. We achieve this by employing the interval number ranking method and incorporating a risk tolerance parameter to reflect the decision-maker’s attitude toward uncertainty.

Given the interval population density $\rho = \left[ {\rho^{ - } ,\rho^{ + } } \right]$ , the transportation risk $\left[ {R_{total}^{ - } ,R_{total}^{ + } } \right]$ in Eq. (8) also becomes an interval number , where the lower and upper bounds are obtained by substituting the bounds of the population density:

9 $$R_{total}^{ - } = \sum\limits_{k = 1}^{e} {\sum\limits_{m = 1}^{{n_{k} }} {\sum\limits_{L = m}^{{n_{k} }} {\frac{{h_{{L_{j}^{k} }} }}{W}} \times P_{{L_{j - 1}^{k} L_{j}^{k} }} \times } } d_{{L_{j - 1}^{k} L_{j}^{k} }} \times \left( {2R \times d_{{L_{j - 1}^{k} L_{j}^{k} }} + \pi \times R^{2} } \right) \times \rho^{ - } ,j \in V,k \in K,$$

10 $$R_{total}^{ + } = \sum\limits_{k = 1}^{e} {\sum\limits_{m = 1}^{{n_{k} }} {\sum\limits_{L = m}^{{n_{k} }} {\frac{{h_{{L_{j}^{k} }} }}{W}} \times P_{{L_{j - 1}^{k} L_{j}^{k} }} \times } } d_{{L_{j - 1}^{k} L_{j}^{k} }} \times \left( {2R \times d_{{L_{j - 1}^{k} L_{j}^{k} }} + \pi \times R^{2} } \right) \times \rho^{ + } ,j \in V,k \in K,$$

A critical step in this transformation is to define a single, crisp risk value for optimization. We introduce a risk tolerance parameter, $\theta_{ij} \in \left[ {0,1} \right]$, which acts as a weighting factor between the best-case (minimum) and worst-case (maximum) risk scenarios for each arc $\left( {i,j} \right)$.

The deterministic transportation risk is then calculated as a convex combination of the lower and upper risk bounds:

11 $$R = (1 - \theta_{ij} ) \times R_{total}^{ - } + \theta_{ij} \times R_{total}^{ + } ,$$

Justification and Interpretation of the Risk Tolerance Parameter $\theta_{ij}$:

$\theta_{ij} = 0$: The model is optimistic, considering only the lower bound of the risk $\left( {R_{total}^{ - } } \right)$. This represents a risk-seeking or highly cost-prioritizing strategy, which may underestimate potential dangers.

$\theta_{ij} = 1$: The model is pessimistic, considering only the upper bound of the risk $\left( {R_{total}^{ + } } \right)$. This represents a highly conservative or safety-first strategy, potentially leading to overly cautious and expensive routes.

$0 < \theta_{ij} < 1$: The model seeks a balanced solution, weighing both optimistic and pessimistic outcomes. The value of $\theta_{ij}$ directly reflects the decision-maker’s willingness to bear risk. A higher value indicates greater risk aversion.

Furthermore, to ensure that the solution is robust against excessive risk realizations, we define an acceptable deviation threshold Δ. This constraint ensures that the worst-case risk for the chosen path does not exceed a predefined limit, adding an extra layer of decision-maker control:

12 $$\max \left( {R_{total}^{ + } - R} \right) \le \Delta = \psi \cdot R_{total}^{ + } ,$$

#### Logistics distribution cost analysis

Within the framework of the issue of transporting dangerous goods by EVs, the total cost of EVs engaged in logistics distribution activities mainly includes vehicle fixed usage costs, charging costs, and additional costs incurred due to failure to arrive within the customer-specified time window.

1. Vehicle fixed costs.

We define the vehicle fixed cost as $C_{fixed}$, which covers the vehicle purchase cost, delivery personnel salaries, and fixed expenses for maintaining vehicle operation. The expression of $C_{fixed}$ is

13 $$C_{fixed} = C_{buy} + C_{veh} \sum\limits_{k \in K} {\sum\limits_{j \in V \cup F} {x_{0j}^{k} } } ,j \in V,k \in K,$$

(2) Charging costs.

The charging cost $C_{ch\arg e}$ of EVs is proportional to the replenished power, which is determined by the driving distance and the power consumption coefficient per unit distance. This paper refers to Literature^20^ to obtain the power consumption coefficient per unit distance $b_{ij}$ for EVs. That is

14 $$b_{ij} = \phi^{d} \varphi^{d} \left( {\frac{{\left( {g\sin \theta \left( t \right) + gf_{s} \cos \theta \left( t \right)} \right)\left( {G + w_{ij} } \right)}}{3600} + \frac{{f_{a} D\rho_{a} v_{t}^{2} }}{76140}} \right),$$

In the formula, $\phi^{d}$ represents the output efficiency parameter of the drive motor. $\varphi^{d}$ represents the output efficiency parameter of the battery. $\theta \left( t \right)$ is the road slope. $G$ is the self-weight of the electric logistics vehicle. $w_{ij}$ is the cargo weight carried by the vehicle at this time. $g$ is the acceleration due to gravity. $f_{s}$ is the rolling resistance coefficient. $f_{a}$ is the air resistance coefficient. $D$ is the vehicle windward area. $\rho_{a}$ is the air density. And $v_{t}$ is the vehicle speed at this time.

Based on the above, the charging cost $C_{ch\arg e}$ is

15 $$C_{ch\arg e} = C_{veh} \sum\limits_{k \in K} {\sum\limits_{{i \in V_{0} \cup F}} {\sum\limits_{{j \in V_{N} \cup F}} {C_{pelec} b_{ij} d_{ij} x_{ij}^{k} } } } ,\forall (i,j) \in A,k \in K,$$

(3) Time window penalty costs.

When the delivery vehicle arrives outside the customer’s acceptable time window, additional costs will be incurred. Early arrival will result in waiting costs (with a unit time cost of $C_{wait}$). Late arrival will generate penalty costs (with a unit time cost of $C_{penal}$). If the customer’s acceptable time window is $\left[ {T_{i}^{1} ,T_{i}^{2} } \right]$, the calculation formula for this cost $C_{time}$ is as follows

16 $$C_{time} = \sum\limits_{k \in K} {\sum\limits_{i \in V} {\left( {C_{wait} \max \left\{ {T_{i}^{1} - t_{i}^{k} ,\left. 0 \right\}} \right. + C_{penal} \max \left\{ {t_{i}^{k} - T_{i}^{2} } \right.,\left. 0 \right\}} \right)} } ,i \in V,k \in K,$$

### Customer average satisfaction

Customer satisfaction is a key indicator for measuring the timeliness and effectiveness of logistics transportation. The traditional “hard” time window model treats any early or late arrival as a complete service failure, which is often too rigid for real-world scenarios. Conversely, a simple “soft” time window only differentiates between on-time and late, without capturing the varying degrees of satisfaction within the acceptable window.

To more realistically and subtly characterize customer satisfaction, this paper adopts a trapezoidal fuzzy membership function to model satisfaction levels. This approach introduces two time windows for each customer:

The Acceptable Time Window $\left[ {T_{i}^{1} ,T_{i}^{2} } \right]$: This is the outer time range within which the customer can still be served, but satisfaction may be less than ideal. Arrivals outside this window are considered unacceptable, resulting in zero satisfaction.

The Ideal Time Window $\left[ {T_{i}^{3} ,T_{i}^{4} } \right]$: This is a stricter, preferred time range nested within the acceptable window. Arrivals within this interval represent the most convenient or ideal service time for the customer, granting full satisfaction.

The relationship between these windows is: $T_{i}^{1} \le T_{i}^{3} \le T_{i}^{4} \le T_{i}^{2}$.

Let $t_{i}$ be the actual arrival time of the vehicle at customer $i$. The customer satisfaction $S_{i} \left( {t_{i} } \right)$ is defined by the following membership function:

17 $$S_{i} (t_{i} ) = \left\{ {\begin{array}{*{20}c} {1 - \left( {\frac{{T_{i}^{3} - t_{i} }}{{T_{i}^{3} - T_{i}^{1} }}} \right)^{\beta } , \, T_{i}^{1} { < }t_{i} < T_{i}^{3} ,\forall i \in V \cup F,k \in K,} \\ {1, \, T_{i}^{3} \le t_{i} \le T_{i}^{4} ,\forall i \in V \cup F,k \in K,} \\ {1 - \left( {\frac{{t_{i} - T_{i}^{4} }}{{T_{i}^{2} - T_{i}^{4} }}} \right)^{\beta } ,T_{i}^{4} < t_{i} < T_{i}^{2} ,\forall i \in V \cup F,k \in K,} \\ {0, \, t_{i} \ge T_{i}^{2} ,\forall i \in V \cup F,k \in K.} \\ \end{array} } \right.,$$

### Interpretation of the membership function and parameters

Core $\left[ {T_{i}^{3} ,T_{i}^{4} } \right]$: arrival within this interval yields maximum satisfaction $S_{i} (t_{i} ) = 1$.

Support $\left[ {T_{i}^{1} ,T_{i}^{2} } \right]$: Arrival within this interval but outside the core yields a satisfaction between 0 and 1. The satisfaction decreases as the arrival time moves further away from the ideal window.

Time-Sensitivity Coefficient $\beta$: This parameter ($\beta > 0$) controls the shape of the satisfaction decay. A linear decay is achieved with $\beta = 1$. A value of $\beta > 1$ makes the function convex, indicating that customers are more tolerant of small deviations but become sharply dissatisfied as the arrival time approaches the boundaries of the acceptable window ($T_{i}^{1} orT_{i}^{2}$). A value of $0 < \beta < 1$ makes the function concave, indicating that customers are highly sensitive to even small deviations from the ideal window. This study sets $\beta$ to 0.8, based on Reference^20^. This choice models a high initial sensitivity to schedule deviations.

Illustrative example: 

Suppose a customer has an acceptable time window of $\left[ {T_{i}^{1} ,T_{i}^{2} } \right]$ = [7:00, 10:00] and an ideal time window of $\left[ {T_{i}^{3} ,T_{i}^{4} } \right]$ = [8:00, 9:00].

If the vehicle arrives at 8:30 (within the ideal window), $S_{i} = 1$.

If it arrives at 7:20, the earliness is 8:00—7:20 = 40 min out of a possible 8:00—7:00 = 60 min. With $\beta = 0.8$, satisfaction is $1 - \frac{40}{{60}} \times 0.8 \approx 0.38$.

If it arrives at 9:30, the lateness is 9:30—9:00 = 30 min out of a possible 10:00—9:00 = 60 min. Satisfaction is $1 - \frac{30}{{60}} \times 0.8 \approx 0.57$.

Arrivals at or before 7:00, or at or after 10:00, result in $S_{i} = 0$.

The overall objective for the optimization model is to maximize the average customer satisfaction across all customers:

18 $$Z_{3} = \frac{1}{n}\sum\limits_{i = 1}^{n} {S_{i} } \left( {t_{i} } \right),$$

### Model establishment

The multi-objective model for EV HMT route optimization in uncertain environments is established as follows:

19 $$Z_{1} = \min R,$$

20 $$Z_{2} = \min \sum\limits_{k \in K} {\left( {C_{fixed} + C_{ch\arg e} + C_{time} } \right)} ,$$

21 $$Z_{3} = \max \frac{1}{n}\sum\limits_{i = 1}^{n} {S_{i} } \left( {t_{i} } \right),$$

22 $$R_{k} \le R_{th} ,k = 1,2, \cdots ,e,$$

23 $$p_{ij} \cdot x_{{_{ij} }}^{k} \le P_{th} ,\forall (i,j) \in A,$$

24 $$r_{i} - r_{j} + N \cdot x_{ij}^{k} \le N - 1,\forall i,j \in V,i \ne j,k \in K,$$

25 $$1 \le r_{i} \le N,\forall i \in V,$$

26 $$\sum\limits_{{i \in V_{0} \cup F}} {x_{ij}^{k} = } \sum\limits_{{i \in V_{0} \cup F}} {x_{ji}^{k} } ,\forall j \in V \cup F,k \in K,$$

27 $$\sum\limits_{j \in V} {x_{0j}^{k} } = \sum\limits_{i \in V} {x_{i0}^{k} } = 1,\forall k \in K,$$

28 $$t_{j}^{k} \ge t_{i}^{k} + w_{i}^{k} + s_{i} + t_{ij} - M \cdot \left( {1 - x_{ij}^{k} } \right),\forall i,j \in V_{0} \cup F,i \ne j,k \in K,$$

29 $$t_{i,wait}^{i} = \max \left\{ {0,} \right.\left. {\left[ {T_{i}^{1} - t_{i}^{k} } \right]} \right\},\forall i \in V,$$

30 $$w_{oj} = W,B_{0} = B,$$

31 $$w_{ij} \le W \cdot x_{ij}^{k} ,\forall \left( {i,j} \right) \in A,k \in K,$$

32 $$B_{j}^{k} \le B_{i}^{k} - b_{ij} \cdot d_{ij} \cdot x_{ij}^{k} + B \cdot \left( {1 - x_{ij}^{k} } \right),\forall i \in V,\forall j \in V,i \ne j,k \in K,$$

33 $$B_{j}^{k} = B_{i}^{k} - b_{ij} \cdot d_{ij} \cdot y_{ij}^{k} + u_{j}^{k} ,\forall i \in V_{0} \cup V,j \in F,k \in K$$

34 $$0 \le u_{j}^{k} \le B - \left( {B_{i}^{k} - b_{ij} \cdot d_{ij} \cdot y_{ij}^{k} } \right),\forall i \in V_{0} \cup V,j \in F,k \in K$$

35 $$B_{i}^{k} - b_{ij} \cdot d_{ij} \cdot x_{ij}^{k} \ge 0,\forall \left( {i,j} \right) \in A,k \in K$$

36 $$0 \le B_{i}^{k} \le B,\forall i \in V_{0} \cup V \cup F,k \in K$$

Equations (19)-(21) are the optimization objective functions of this model. Equation (22) represents the risk constraint of a certain distribution path. Equation (23) represents the accident probability threshold. Equation (24) ensures that if vehicle k visits node $j$ from node $i$,Then the access order $r_{j}$ must be strictly greater than $r_{i}$. Equation (25) defined the boundaries of auxiliary variables $r_{i}$. Equation (26) ensure that the traffic flow is conserved for each vehicle when entering and leaving any node. Equation (27) Ensure that each vehicle k departs from the distribution center (0) and eventually returns to the distribution center. Equation (28) Ensure that the arrival time at node $j$ is not earlier than the departure time from the previous node $i$ plus the travel time,M is a sufficiently large constant. Equation (29) represents the possible waiting time range during distribution. Equation (30) shows the vehicle starting under full load conditions with a fully charged battery. Equation (31) indicates that the vehicle’s load at any node must not exceed the maximum vehicle load. Equation (32) is the electricity balance constraint,It ensures the continuity of power consumption along the path. Equation (33) indicates that when vehicle k travels from node i to charging station j for recharging, its battery level is replenished after consumption. Equation (34) indicates that the amount of charging at any charging station cannot exceed the remaining capacity of the battery. Equation (35) indicates that after the vehicle travels any arc segment, its remaining battery must be non-negative to ensure it can reach the next node. Equation (36) indicates that the vehicle’s battery level at any node cannot exceed its maximum battery capacity.

### Design of multi-objective genetic algorithm

**Fig. 2:**
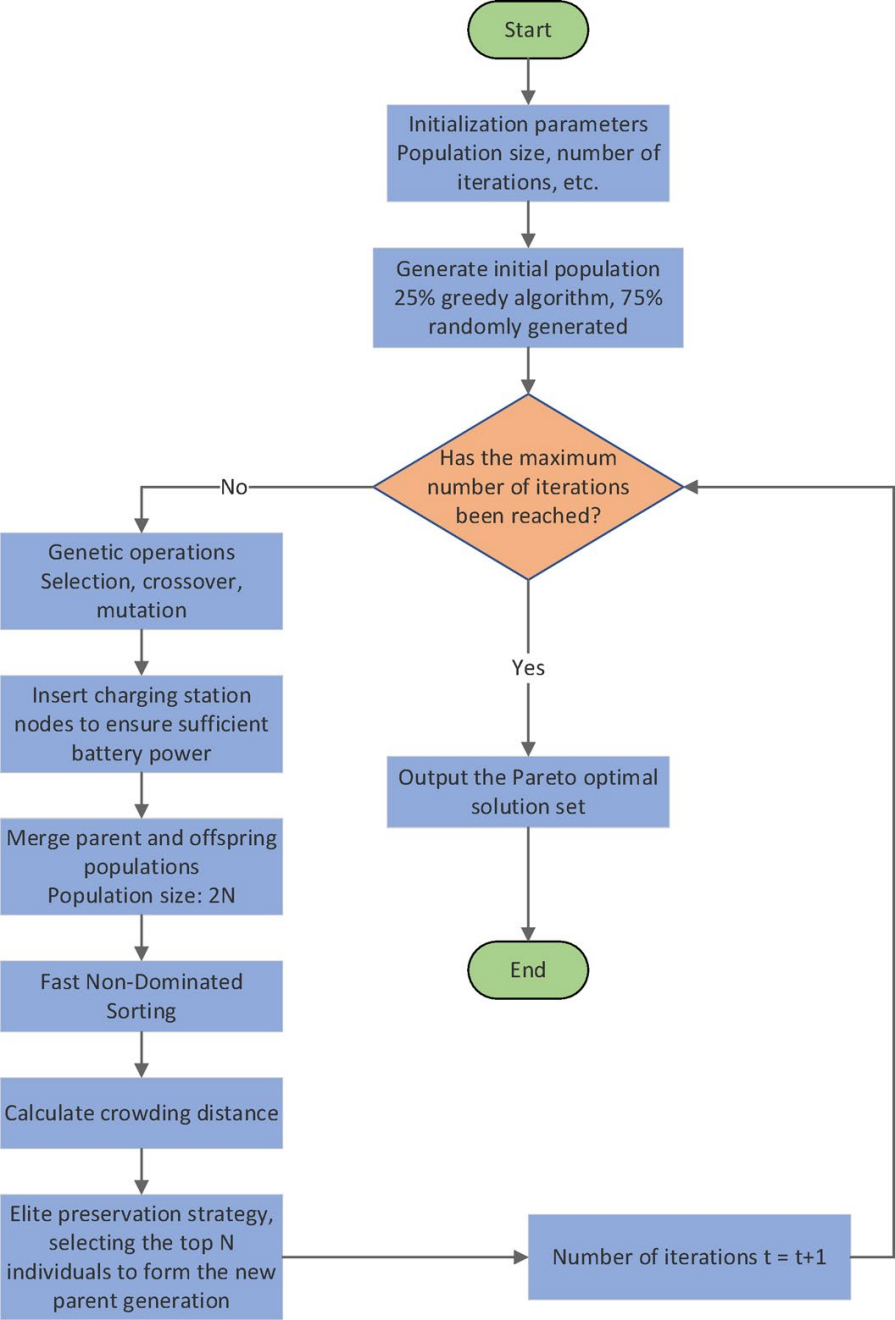
Algorithm flowchart.

The solution to multi-objective optimization problems mainly relies on two methods: exact algorithms and heuristic algorithms. The HMT VRP with time windows belongs to the NP-Hard problem category. The computational complexity of such problems usually increases exponentially with the increase in customer scale, and large-scale VRPs can only be solved by designing effective heuristic algorithms. The NSGA-II performs superiorly in solving VRPs in terms of solution accuracy and computation time. This paper combines the NSGA-II algorithm with the greedy algorithm to design an improved NSGA-II algorithm for solving the model in this paper, namely H-NSGA-II. The process of the algorithm is shown in Fig 2.

### Chromosome construction

**Fig. 3:**
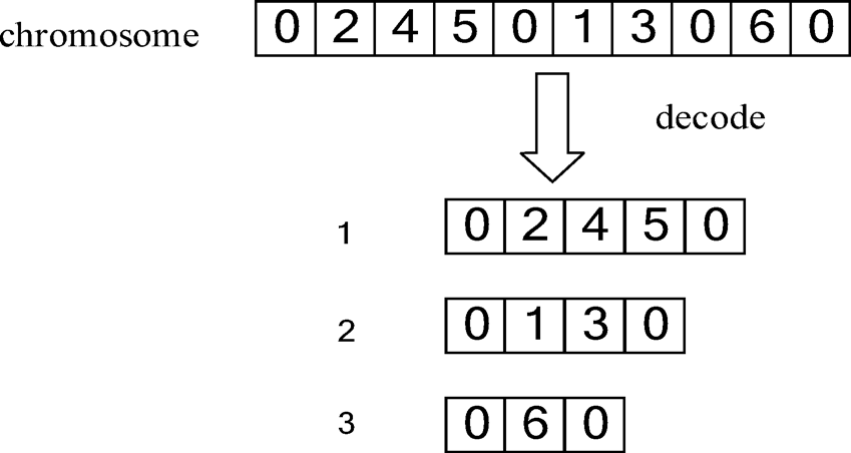
Chromosome coding and decoding.

This paper employs a natural number coding based on path representation for chromosome design. The distribution center is numbered ‘0’, and customers are numbered 1, 2, ..., n. A chromosome is a sequence comprising all customer nodes and the distribution center ‘0’ which acts as a route separator. All vehicles depart from and return to the distribution center. The length of a chromosome is variable, dependent on the number of vehicles K used in the solution. Specifically, for a solution utilizing K vehicles, the chromosome length is n + k + 1 (including the starting and terminating ‘0’). In our implementation, we set a sufficiently large maximum chromosome length to accommodate all potential routes For example, if a distribution center has 3 vehicles serving 6 customers, one chromosome can be expressed as (0, 2, 4, 5, 0, 1, 3, 0, 6, 0), and the chromosome coding and decoding are shown in Fig 3. The aforementioned chromosome encoding defines the skeleton of a solution. Next, the decoding process translates this skeleton into a complete, evaluable distribution plan.

### Solution decoding and objective evaluation

Decoding a chromosome into an evaluable distribution plan represents one of the most critical steps in heuristic algorithms. This section elaborates on the derivation of a complete routing solution from the chromosome structure, the procedures for ensuring feasibility with respect to key constraints, and the methodology for computing the corresponding multi-objective function values.

The decoding process converts the chromosome sequence into concrete vehicle routes while performing simultaneous constraint validation and repair. The logic for handling core constraints is outlined as follows:

1. Vehicle load constraints: load is calculated in real-time during customer assignment. If adding a new customer causes the cumulative load to exceed vehicle capacity, the current route is terminated immediately. The vehicle returns to the depot (node 0), and a new route is initiated. This process directly determines the positions of the route separator 0, delineating the end of one route and the start of another.
2. Time window constraints: the “earliest arrival, immediate service” rule is adopted. If a vehicle arrives before the earliest service time, it waits until service can begin, incurring a waiting cost. If it arrives within the time window, service starts immediately. Arrivals later than the latest allowable time are deemed infeasible; such solutions are penalized with a very large value to ensure their elimination during evolution. This mechanism establishes the actual arrival and service times, forming the basis for calculating time window penalty costs and customer satisfaction.
3. Battery and charging constraints: the remaining battery level is tracked in real-time as the vehicle traverses its route. If the charge is insufficient to reach the next customer or return to the depot, the nearest feasible charging station between the current location and the next target is inserted into the route. Charging time is incorporated into the total travel time, and charging cost is added to the total cost. This dynamic modification of the path directly influences both temporal and economic objectives.

Once a complete and feasible distribution plan is obtained—where all customers are served and all constraints are satisfied—the three objective function values are computed as follows:

1. Total transportation risk is derived using real-time vehicle load and interval-based uncertain population density for corresponding road segments.
2. Customer satisfaction is calculated per arrival time and fuzzy time window using the defined membership function, then averaged across all customers.
3. Total cost is evaluated based on fixed, charging, and time-window penalty components.

In summary, the decoder serves as the core module that maps a chromosome to a feasible solution. By embedding the constraint-handling rules described above, it guarantees the physical executability of decoded routes. Through simulation of the full distribution plan, it accurately evaluates the three objective functions, thereby providing the essential basis for fitness assessment in the genetic algorithm.

### Population initialization

The quality of the initial population is crucial for the algorithm’s global search capability and convergence speed. To simultaneously introduce high-quality solutions and diversity in the initial stage, this paper adopts a hybrid initialization strategy: 25% of the initial individuals are generated by a greedy algorithm to provide high-quality starting points, while the remaining 75% are generated randomly to ensure population diversity.

The core of the greedy algorithm lies in its customer selection strategy. To achieve this, we designed a composite cost function that translates the model’s three global objectives (transportation risk, transportation cost, and customer satisfaction) into an executable local decision criterion. The function is defined as follows:

37 $${\mathrm{Cos}} t\left( {i,j} \right) = \alpha \cdot \frac{{d_{ij} }}{\max \left( d \right)} + \beta \cdot \frac{{Time_{j} }}{{\max \left( {Time} \right)}} + \gamma \cdot \frac{{h_{j} }}{H},\forall (i,j) \in A,j \in V,$$

Here, $d_{ij}$ is the distance, $Time_{j}$ represents the time urgency of customer $j$, and $h_{ij}$ is its demand. The weighting coefficients are set as α = 0.4, β = 0.5, and γ = 0.1 to prioritize the satisfaction of time window constraints. This function ensures that each step tends to select the customer who is optimal in terms of distance, time, and load combined.

**Fig. 4:**
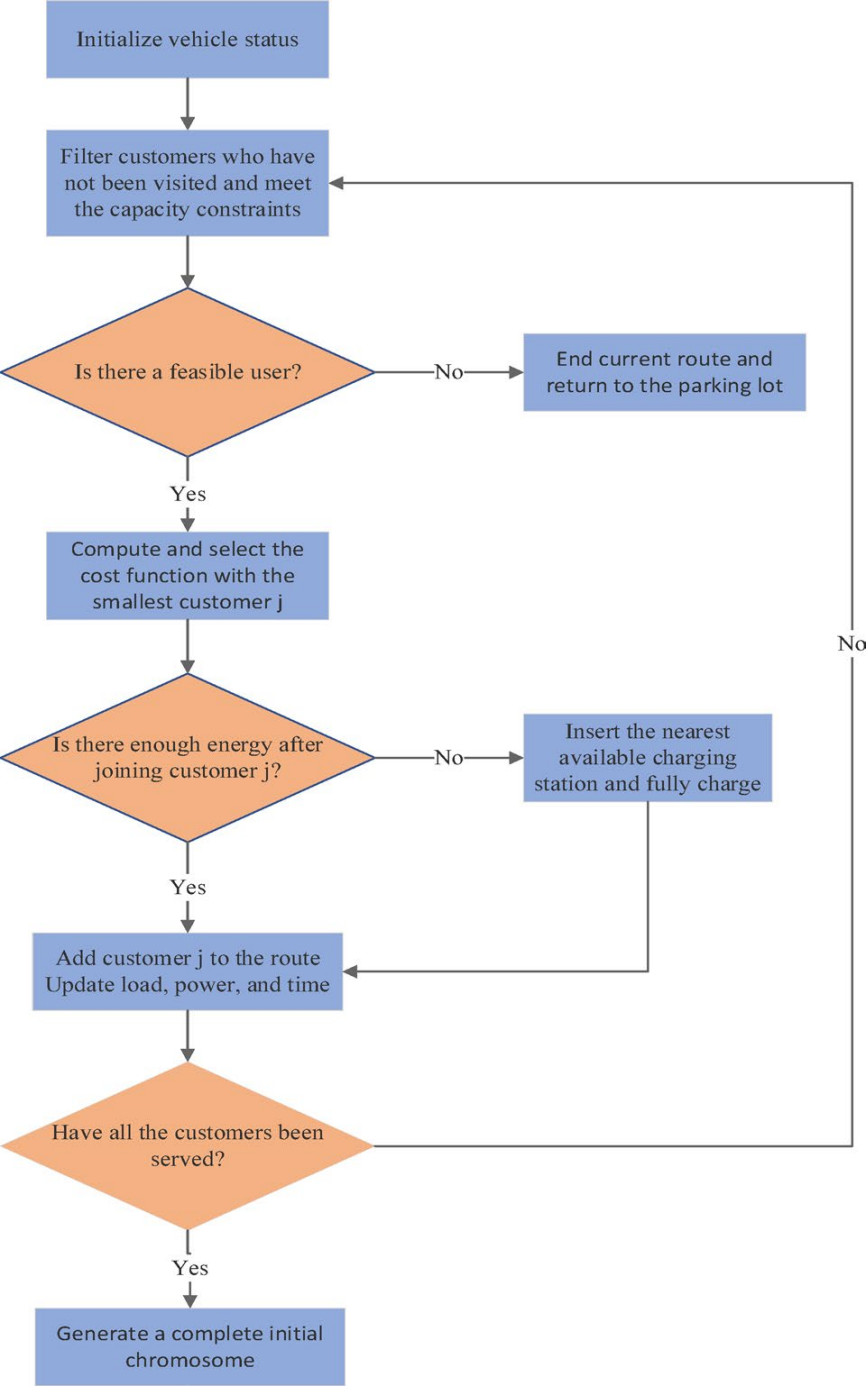
Flowchart of the greedy algorithm for initial solution construction.

Based on this cost function, the flowchart of the greedy algorithm for constructing a single route is shown in Fig 4, and its steps can be summarized as follows:

1. Start from the depot and initialize the vehicle state.
2. Filter feasible candidate customers that satisfy the hard constraints.
3. Use the aforementioned cost function to select the next best customer.
4. Perform an energy check, inserting the nearest charging station if insufficient.
5. Serve the customer and update the vehicle state.
6. Repeat the above process until the current route is terminated, then dispatch a new vehicle until all customers are served.

This method efficiently generates feasible solutions that are highly consistent with the global optimization objectives, laying a solid foundation for subsequent evolutionary operations.

### Crossover and mutation operations

To ensure population diversity and individual effectiveness, this paper uses a position-based crossover method. Several discontinuous positions are randomly selected on two parent chromosomes, and the genes selected from parent chromosome 1 are copied to the same positions in offspring chromosome 1. The missing genes in offspring 1 are filled by the genes from parent chromosome 2 in order and one-to-one correspondence, with duplicate genes in offspring chromosome 1 removed. Offspring chromosome 2 is generated in the same way.

**Fig. 5:**
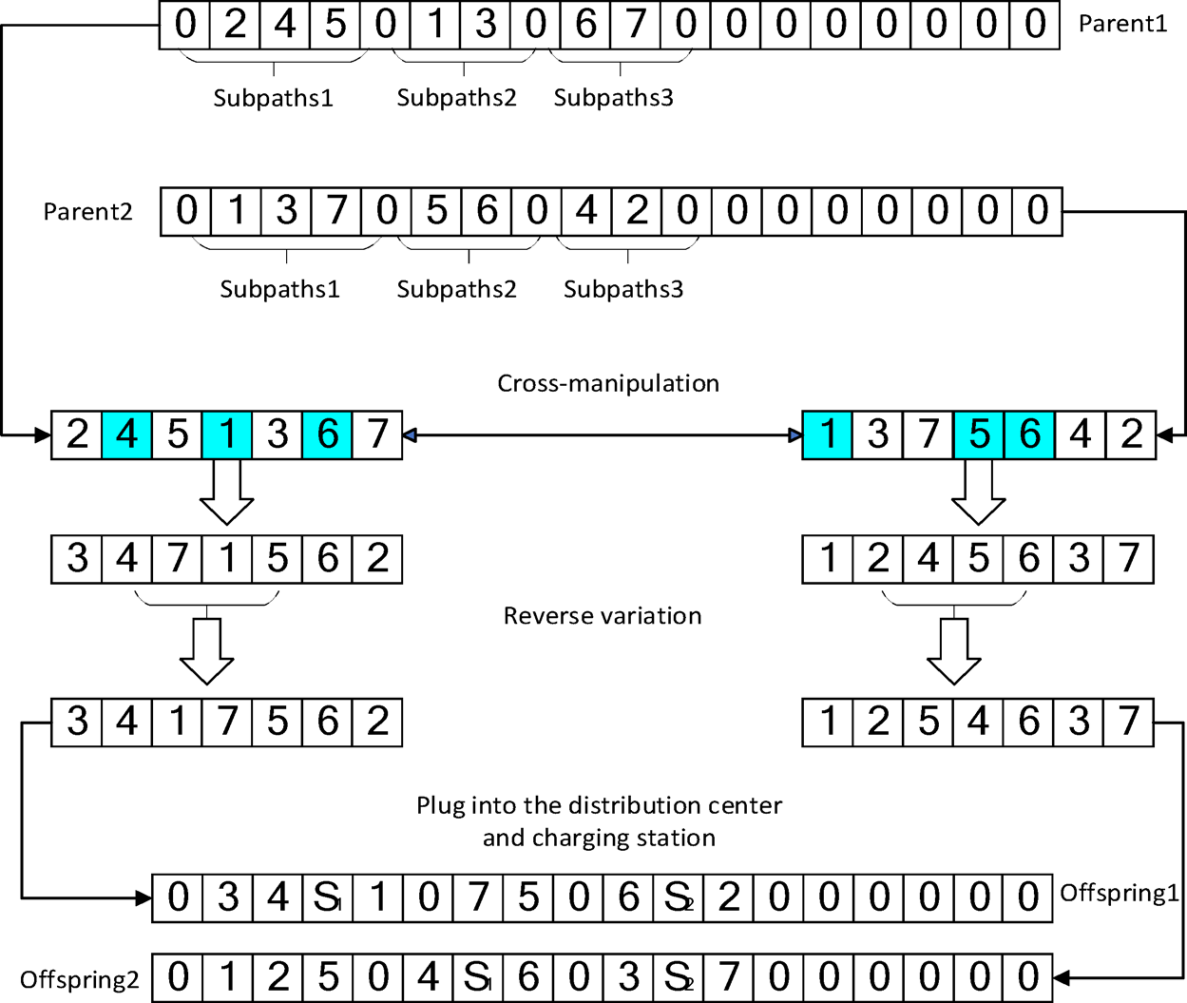
Schematic diagram of crossover and mutation.

In mutation operations, three different neighborhood operators (swap, reverse, and insert) are designed. Based on the individuals obtained from crossover, genes are modified with probabilities of 0.2, 0.5, and 0.3 respectively to explore more possibilities and help the algorithm escape local optima. The diagram of crossover and mutation is shown in Fig 5.

**Fig. 6:**
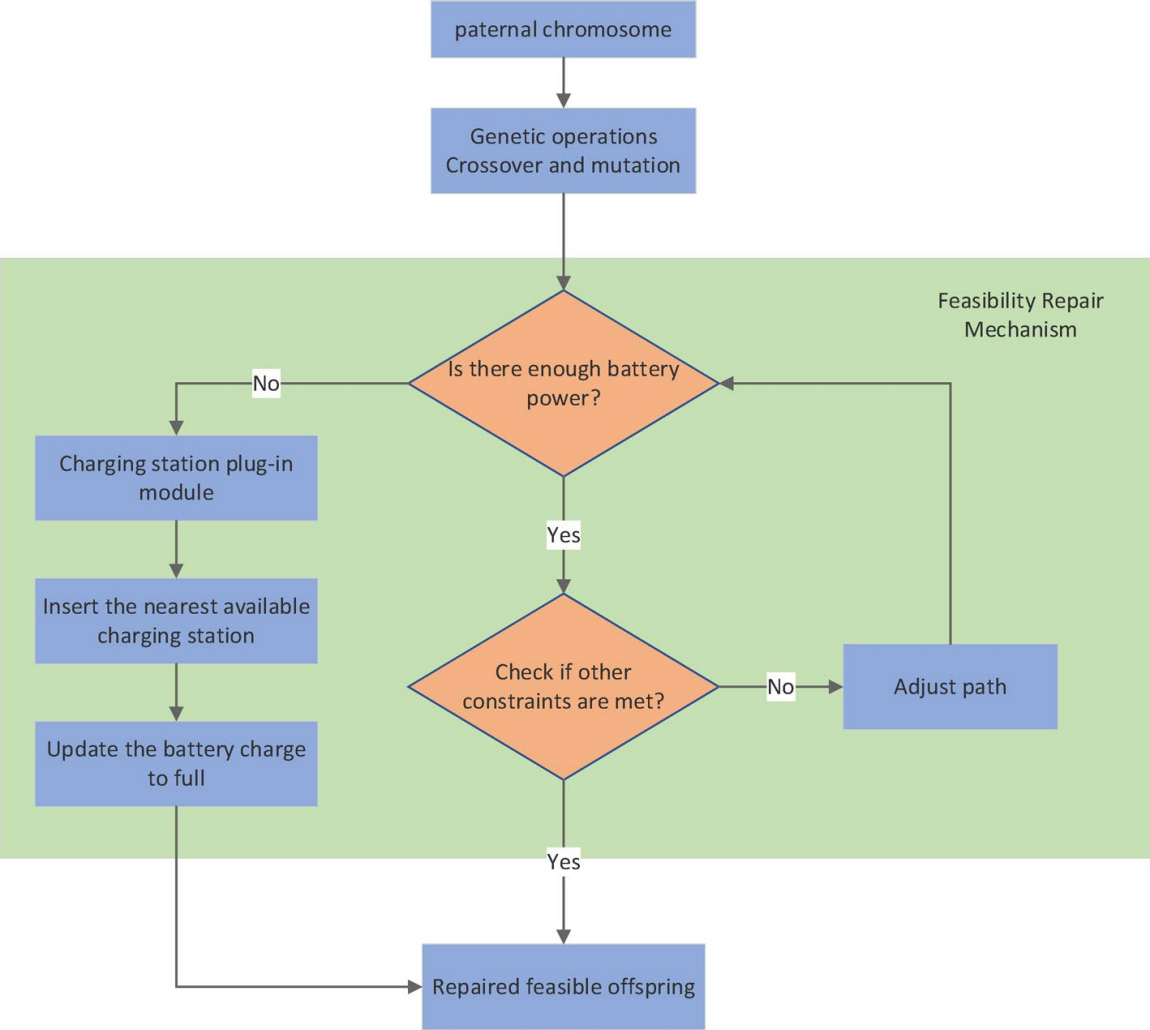
A schematic diagram of the genetic manipulation and feasible repair process.

Following crossover and mutation operations, a feasibility repair mechanism is activated to insert charging stations and ensure solution feasibility. A charging station is inserted into a route if the remaining battery level upon leaving a node is insufficient to serve the next customer or return to the depot. The selected charging station is the nearest one that does not violate the vehicle’s time window constraints. After charging, the vehicle’s battery is reset to full capacity. This mechanism guarantees that all offspring solutions considered for the next generation are feasible regarding energy and time window constraints. A schematic diagram of the genetic manipulation and feasible repair process is shown in Fig 6.

### Elite retention strategy

To ensure that the algorithm retains the optimal individuals during the iteration process, this strategy adopts the following steps:

1. Population merging. Merge the parent population containing N individuals with the offspring population generated through crossover and mutation operations (also containing N individuals) to form a combined population of size 2N.
2. Non-dominated sorting. Use the fast non-dominated sorting method to stratify the combined population, dividing individuals into different non-dominated levels (Front 1, Front 2, ...).
3. Crowding distance calculation and sorting. For individuals at the same non-dominated level, calculate their crowding distance and sort them based on this metric (typically, individuals with higher crowding distance are prioritized).
4. Elitist selection. Based on the non-dominated level of individuals (with higher priority) and the crowding distance sorting within the same level, sequentially select the top N optimal individuals from the combined population to form the next generation population.

**Fig. 7:**
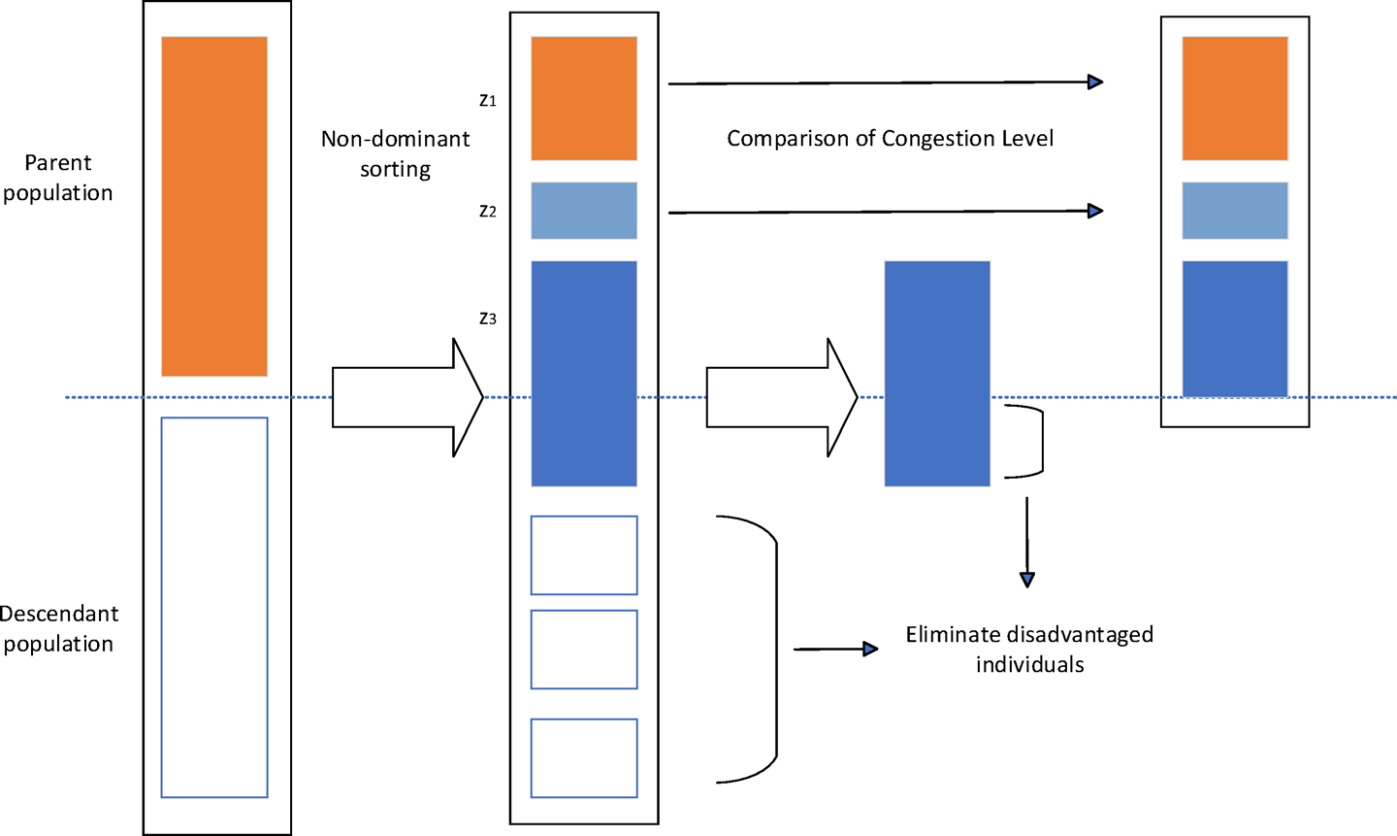
Elite retention strategy.

Fig 7 illustrates the complete process of this elitist retention strategy in one algorithm cycle.

### Analysis of case simulation results

#### Case data and parameter settings

**Fig. 8:**
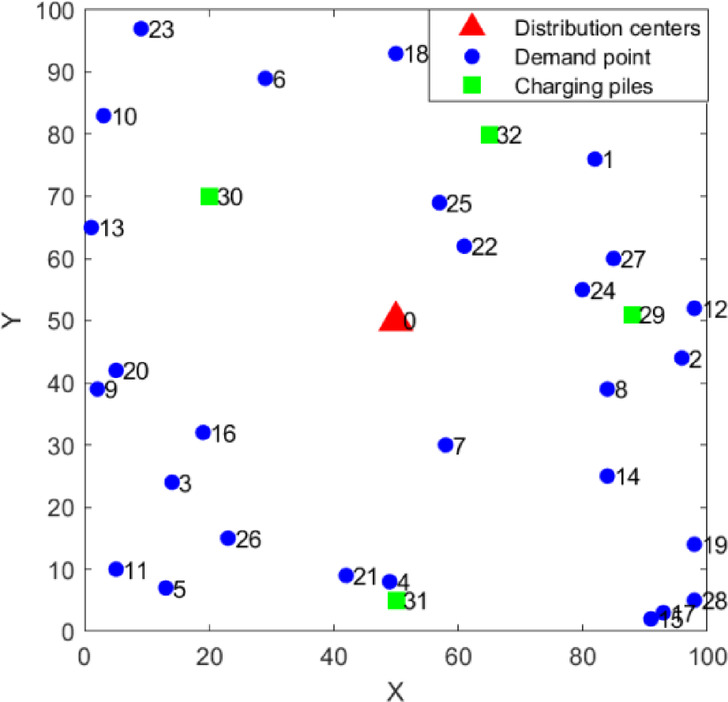
Location map of logistics distribution system.

The test case has 33 nodes, where 0 is the distribution center, 1-28 are customer demand points, and 29-32 are charging piles. Partial logistics distribution information is detailed in Table 3, and the distribution diagram of each node is shown in Fig 8. The algorithm runs on a computer with an Intel Core i5-12450H processor at 2.00 GHz, Windows 11 64-bit operating system, using MATLAB 2023b as the platform.

**Table 3:** Distribution information.

Node	Abscissa	Ordinate	Demand (t)	Fuzzy time window (h)			
				Earliest service time	Latest service time	Earliest departure time	Latest departure time
0	50	50	0	0	0	18	20
1	82	76	0.5	7	7.2	7.6	8
2	96	44	0.5	7.5	8	8.6	9
3	14	24	0.5	6.5	7	8	8.5
4	49	8	0.1	9	9.5	10.6	11
5	13	7	0.1	9.8	10.2	10.9	11.4
6	29	89	0.1	10.5	11	11.8	12.3
7	58	30	0.1	10	10.5	11.5	11.8
8	84	39	0.1	6.1	6.6	7	7.5
9	2	39	0.1	7.5	8	9	9.5
10	3	83	0.1	9.5	10	10.5	11
11	5	10	0.1	10	10.5	11	11.5
12	98	52	0.2	7	7.5	8	9
13	1	65	0.2	8.5	9	9.5	10
14	84	25	0.2	8	8.5	9	9.5
15	91	2	0.2	7.5	8	9	9.5
16	19	32	0.2	7	7.5	8	8.5
17	93	3	0.2	7.5	8	8.5	9
18	50	93	0.3	11	11.5	12.5	13
19	98	14	0.3	7	7.5	8.5	9
20	5	42	0.3	7.5	8	8.7	9.3
21	42	9	0.3	9.5	10	10.8	11.3
22	61	62	0.05	8	8.5	9	9.5
23	9	97	0.05	10	10.5	11	11.5
24	80	55	0.6	6.5	7	8.5	9
25	57	69	0.6	7.5	8	9	9.5
26	23	15	0.5	6.5	7	8.5	9
27	85	60	0.5	6.1	6.5	7	7.5
28	98	5	1	6.7	7.2	8	8.5
29	88	51	0	0	0	18	20
30	20	70	0	0	0	18	20
31	50	5	0	0	0	18	20
32	65	80	0	0	0	18	20

The H-NSGA-II algorithm parameters are set as follows: population size is 120, maximum number of iterations is 500, crossover probability is 0.85, and mutation probability is 0.15. The distance between each logistics node is calculated using Euclidean distance. The vehicle has a weight of 2.5 tons and travels at a constant speed of 40 km/h. Other related parameters are shown in Table 4.

**Table 4:** Relevant calculation parameters.

Parameter	Value	Parameter	Value	Parameter	Value
$R$	400 m	$B$	45 kWh	$\phi^{d}$	1.184692
$\rho$	2000–5000 people per km^2^	$\theta \left( t \right)$	2%	$\varphi^{d}$	1.112434
$\theta_{ij}$	0.5	$C_{buy}$	220 thousand RMB	$f_{a}$	0.7
$P$	0.000001	$C_{veh}$	200 RMB per vehicle	$f_{s}$	0.012
$P_{th}$	0.045	$C_{pelec}$	0.7 RMB per kWh	$g$	9.8 m/s2
$\psi$	0.15	$C_{wait}$	10 RMB per hour	$D$	3.8 m2
$W$	2.5t	$C_{penal}$	40 RMB per hour	$\rho_{a}$	1.2041 kg/m3

### Result ansalysis

**Fig. 9:**
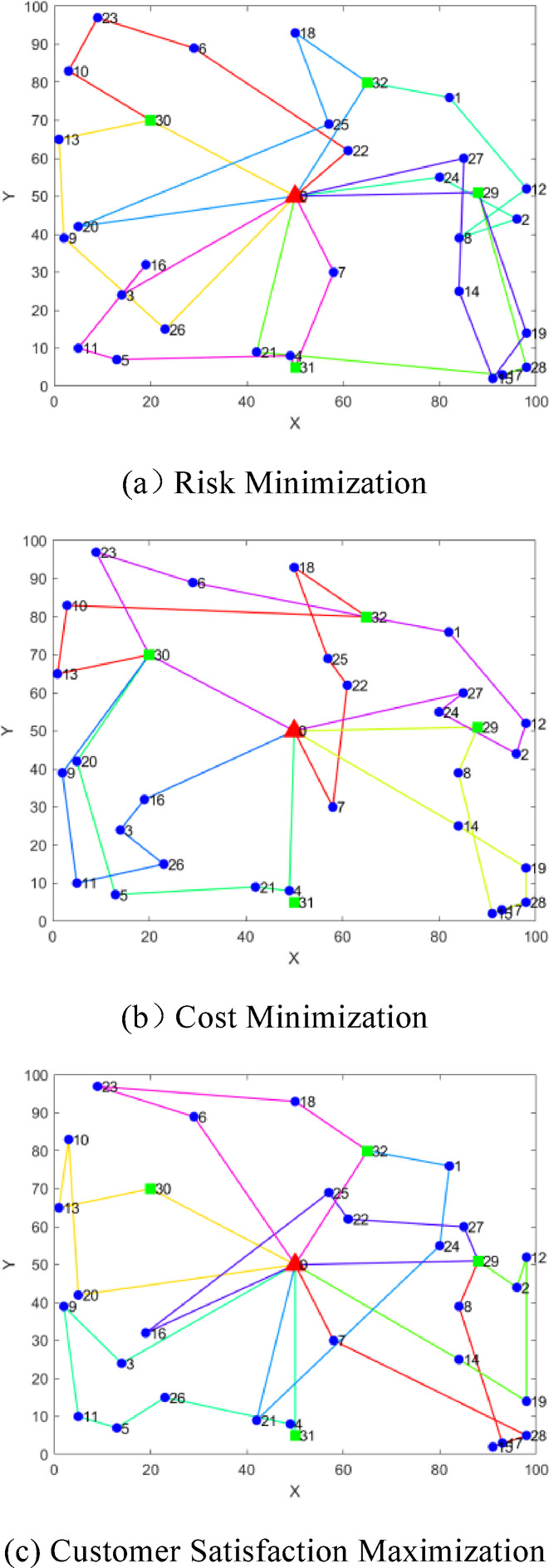
Schematic diagram of path optimization for each objective by improved NSGA-II algorithm.

The optimal HMT route plans for each objective in the Pareto solution set obtained by the algorithm are shown in Table 5, and the specific transportation routes areshown in Fig. 9. Through comparative analysis, the results are as follows: (1)When the transportation risk $Z_{1}$ is optimized, both transportation cost and average customer satisfaction achieve non-dominated solutions, with a moderate number of vehicles deployed. Compared to the optimization of $Z_{2}$ and $Z_{3}$, transportation risk is reduced by 26.75% and 13.12%, respectively. However, transportation cost increases by 24.54% compared to $Z_{2}$ optimization, and average customer satisfaction decreases by 9.34% compared to $Z_{3}$ optimization. (2) When the transportation cost $Z_{2}$ is optimized, transportation risk and average customer satisfaction achieve non-dominated solutions, with the minimum number of vehicles used. Compared to $Z_{1}$ and $Z_{3}$ optimizations, transportation cost is reduced by 19.71% and 27.85%, respectively. However, transportation risk increases by 36.52% compared to $Z_{1}$ optimization, and average customer satisfaction decreases by 23.99% compared to $Z_{3}$ optimization. (3) When the average customer satisfaction $Z_{3}$ is optimized, transportation risk and transportation cost achieve non-dominated solutions, with a moderate number of vehicles deployed. Compared to $Z_{1}$ and $Z_{2}$ optimizations, average customer satisfaction increases by 10.30% and 31.57%, respectively. However, transportation risk increases by 15.11% compared to $Z_{1}$ optimization, and transportation cost increases by 38.59% compared to $Z_{2}$ optimization.

**Table 5:** Optimal values of each objective and path set.

Single optimal solution	Objective function			Path number	Path set
	$Z_{1}$	$Z_{2}$	$Z_{3}$		
$Z_{1}$	0.523	5795.78	0.563	1	0–22-6–23-10-[30]-0
				2	0–26-9–13-[30]-0
				3	0–21-17–28-[29]-0
				4	0–24-2–8-12–1-[32]-0
				5	0–20-25–18-[32]-0
				6	0–27-14–15-19-[29]-0
				7	0–3-16–11-5–4-[31]-7–0
$Z_{2}$	0.714	4653.67	0.472	1	0–7-22–25-18-[32]-10–13-[30]-0
				2	0–14-19–28-17–15-8-[29]-0
				3	0–4-21–5-20-[30]-0
				4	0–16-3–26-11–9-[30]-0
				5	0–27-24–2-12–1-[32]-6–23-[30]-0
$Z_{3}$	0.602	6449.55	0.621	1	0–7-28–15-17–8-[29]-0
				2	0–20-10–13-[30]-0
				3	0–14-19–12-2-[29]-0
				4	0–3-9–11-5–26-4-[31]-0
				5	0–21-24–1-[32]-0
				6	0–16-25–22-27-[29]-0
				7	0–6-23–18-[32]-0

### Pareto solution set distribution and decision support

**Fig. 10:**
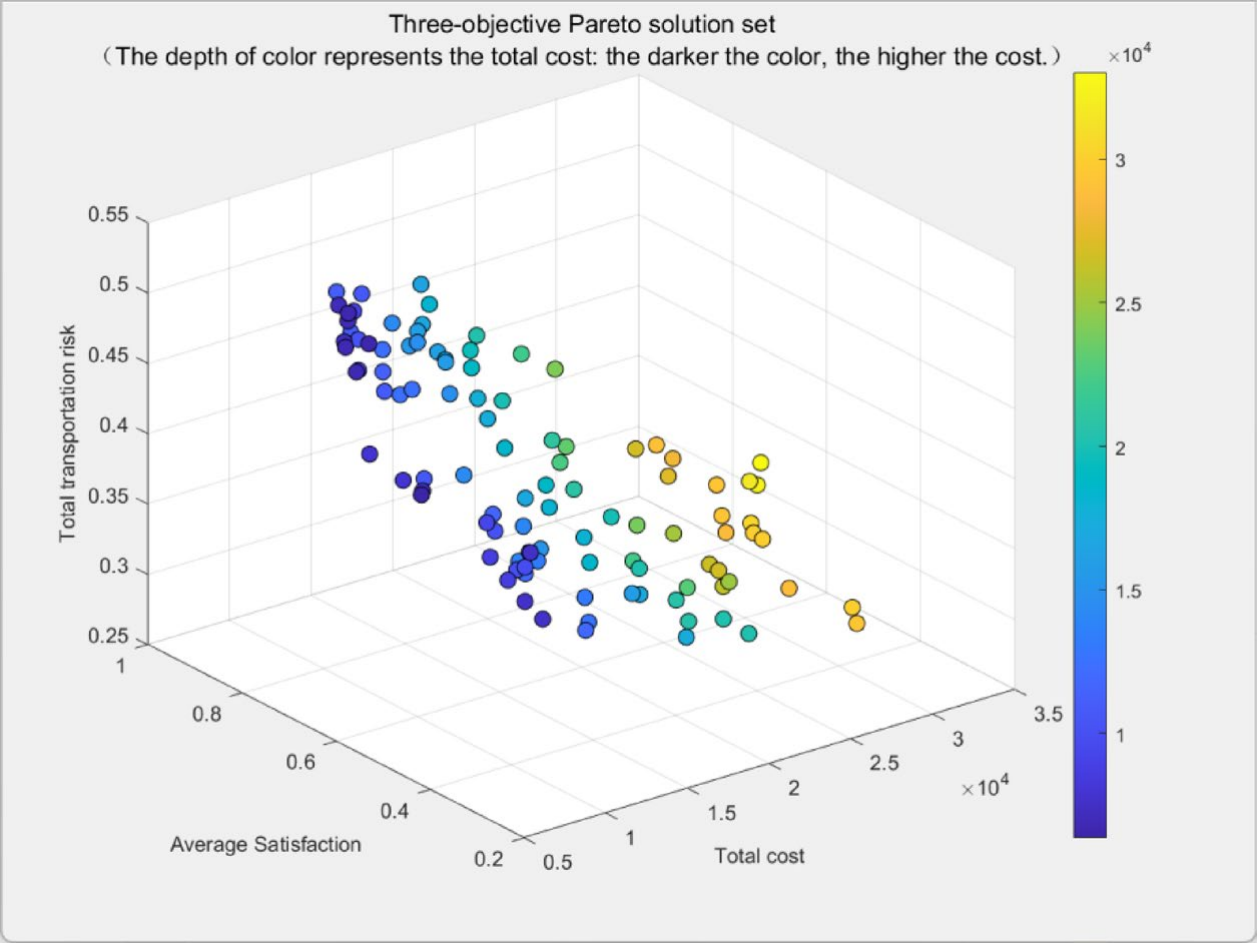
Pareto front distribution plot.

The Pareto solution set derived from the algorithm is presented in Fig 10. Regarding its distribution characteristics, the Pareto solutions in the 3D graph exhibit a wide spread, covering the entire objective space ranging from “low risk—satisfaction—low cost” to “high risk—high satisfaction—high cost”. Notably, the solution density is higher in local regions such as the “medium risk—medium satisfaction—medium cost” interval. This demonstrates that the Pareto front generated by the algorithm possesses excellent diversity and uniformity, offering abundant alternatives for decision-makers with different preferences.

Based on this, corresponding scenario-specific decision-making recommendations are proposed as follows: (1) Risk-priority scenario: Opt for solutions in the purple area (featuring the lowest risk), which requires accepting the trade-off of “lower satisfaction and moderate cost”. This is suitable for scenarios with extremely low risk tolerance, such as hazardous materials transportation. (2) Cost-priority scenario: Choose solutions in the blue area (with the minimum cost), involving a trade-off between “lower satisfaction and moderate risk”. It applies to general transportation scenarios where cost control is a top priority for enterprises. (3) Satisfaction-priority scenario: Select solutions in the green area (offering the highest satisfaction), which entails accepting the trade-off of “higher cost and higher risk”. This is applicable to scenarios with high demands for timeliness and service experience.

**Fig. 11:**
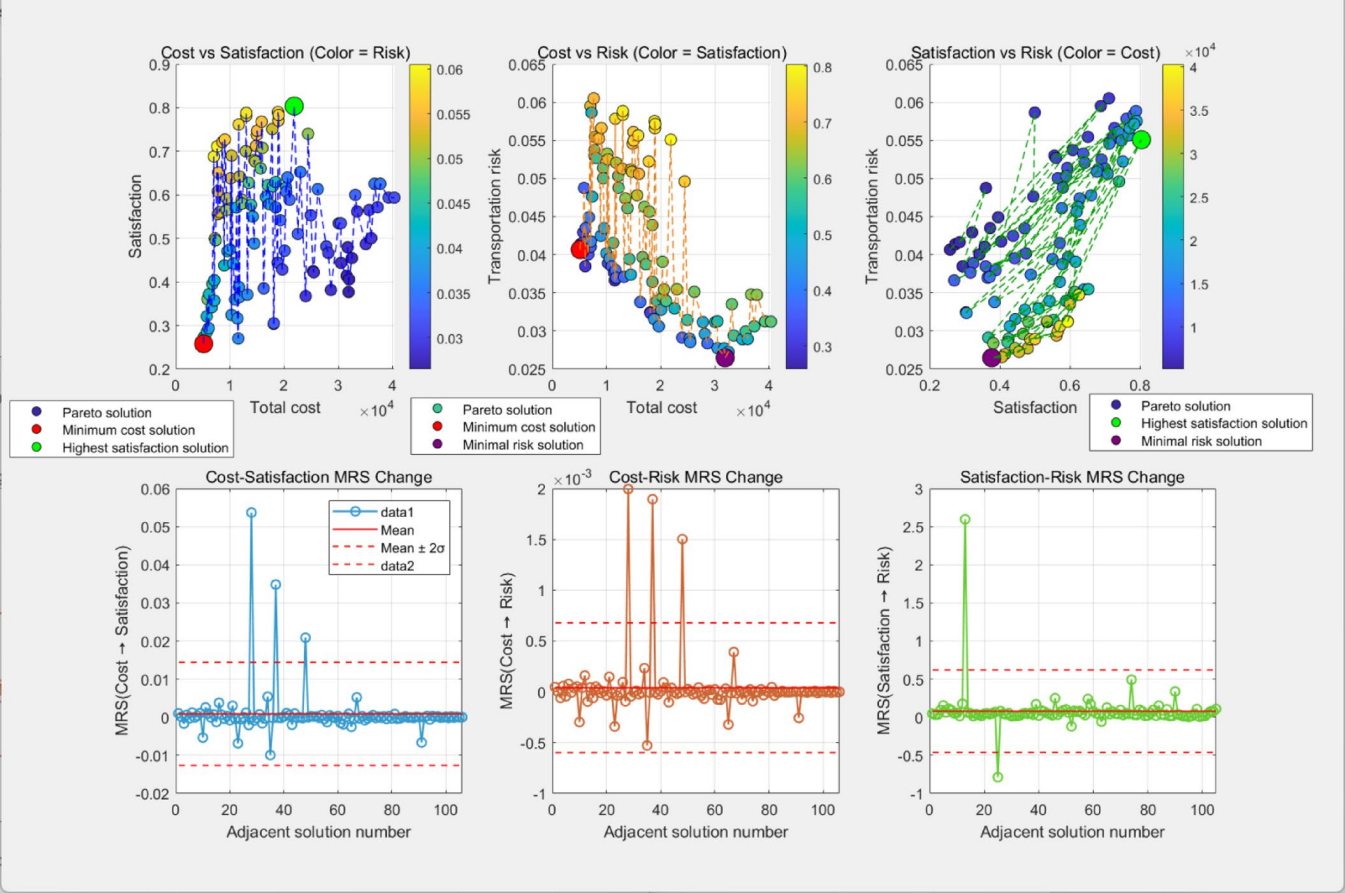
Global conflict and local conflict results.

To further clarify the interaction mechanisms among the three objectives (transportation risk, total cost, and customer satisfaction), this study quantifies their conflict characteristics from both global and local dimensions by combining correlation analysis with the Marginal Rate of Substitution (MRS). All correlation results passed the significance test (p < 0.05), providing precise quantitative support for subsequent decision-making. The relevant results are visualized in Fig 11, and the global conflict analysis is elaborated as follows:

1. Total cost vs. satisfaction: the correlation coefficient is 0.0966, reflecting a weak positive correlation and mild conflict. The trade-off between these two objectives is minimal, indicating substantial potential for synergistic optimization. An increase in total cost is accompanied by a noticeable improvement in satisfaction, as moderate cost investments—such as optimizing route efficiency, enhancing charging support, or reducing waiting times—can effectively elevate service quality without excessive sacrifice of either objective.
2. Total cost vs. transportation risk: the correlation coefficient is -0.6514, representing a moderate negative correlation and moderate conflict. A distinct trade-off exists here: higher total cost leads to a significant reduction in transportation risk, achieved through cost-incurring measures like avoiding high-population-density areas and optimizing vehicle allocation. Improving one objective in this pair necessitates a moderate compromise on the other.
3. Satisfaction vs. transportation risk: the correlation coefficient is 0.6149, indicating a moderate positive correlation and moderate conflict. Greater satisfaction is associated with a notable increase in transportation risk, primarily because meeting time window requirements may involve selecting shorter yet slightly riskier routes. While this conflict is evident, its intensity is not extreme, leaving ample room for refined optimization strategies.

The MRS quantifies the change in one objective when another objective increases by one unit between adjacent Pareto solutions, with the results detailed as follows:

1. Cost → Satisfaction MRS:The average MRS is 0.000882. A positive MRS indicates that increasing cost enhances satisfaction—on average, each unit rise in cost yields a 0.000882 unit gain in satisfaction. Three conflict inflection points were identified in adjacent solution intervals (e.g., intervals 2, 3, 4, 8), where the trade-off between cost and satisfaction changes abruptly. This implies that cost investment within these specific intervals can drive a significant leap in satisfaction.
2. Cost → Risk MRS:The average MRS is 0.000040. A negative MRS signifies that higher cost reduces transportation risk—on average, each additional unit of cost lowers risk by 0.000040 units. Similarly, three conflict inflection points (e.g., intervals 2, 3, 4, 8) were observed, where the marginal effect of cost on risk shifts sharply. These intervals represent the most efficient phases for achieving risk reduction through cost investment.
3. Satisfaction → Risk MRS:The average MRS is 0.079293. A positive MRS confirms that improved satisfaction generally correlates with increased transportation risk—on average, each unit gain in satisfaction raises risk by 0.079293 units. Two conflict inflection points were detected in adjacent solution intervals (e.g., intervals 1, 2, 3), where the trade-off between the two objectives undergoes a significant change. Notably, in certain sub-intervals, satisfaction can be enhanced while simultaneously reducing risk, highlighting the potential for local synergistic optimization

Based on the above analysis, inherent trade-offs are evident in multi-objective optimal route selection: emphasizing both transport safety and high customer satisfaction inevitably drives up total cost, while prioritizing cost control leads to elevated risk and reduced satisfaction. The quantitative results of global and local conflicts clarify the intensity and dynamic characteristics of objective interactions, and the identified decision boundaries and optimization no-go zones provide clear constraints for practical decision-making. Decision-makers can set appropriate target thresholds based on scenario-specific requirements (e.g., risk-priority, cost-priority, or satisfaction-priority) and select the optimal solution from the Pareto set to achieve a balanced multi-objective outcome while satisfying constraints.

### Algorithm performance analysis

Based on the benchmark case in this study, both algorithms were tested under identical conditions, with the optimal results summarized in Table 6. Data analysis clearly demonstrates that the H-NSGA-II outperforms the standard NSGA-II across all key performance metrics. Specifically, the improved algorithm reduces the optimal transportation risk by 14.40% and the optimal transportation cost by 12.81%, while increasing the optimal average customer satisfaction by 13.53%. Additionally, the H-NSGA-II shortens the computation time by 56 seconds, yields a greater number of Pareto solutions, and improves the hypervolume index from 0.685 to 0.752. These results confirm that the improved NSGA-II exhibits superior performance in solution accuracy, convergence speed, and solution set diversity, making it more effective for supporting decision-making in hazardous materials transportation route planning.

**Table 6:** Optimal results of different algorithms.

	NSGA-II	Improved NSGA-II
Optimal transportation risk	0.611	0.523
Optimal transportation cost	5336.78	4653.67
Optimal average customer satisfaction	0.547	0.621
Algorithm runtime(s)	218	162
Number of Pareto solutions	82	105
Supervolume Index	0.685	0.752

### Sensitivity analysis

**Fig. 12:**
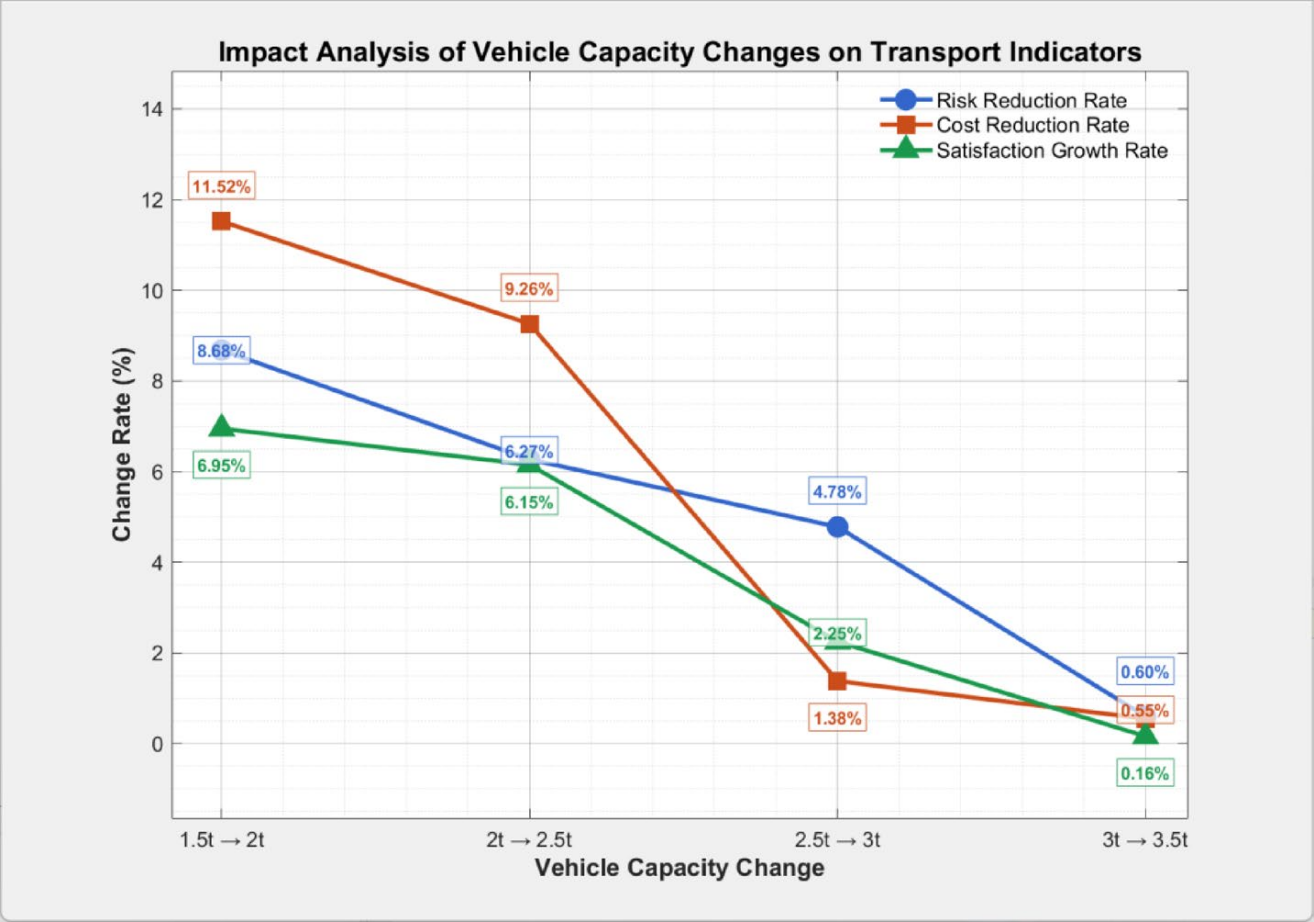
Rate of change of the optimization objective under different vehicle capacity increments.

To explore the impact of vehicle capacity on the findings of this study, comparative experiments were conducted. With algorithm parameters kept consistent, vehicle capacities were set to 1.5t, 2t, 2.5t, 3t, and 3.5t respectively, focusing on the optimization objectives and the number of vehicles used. Detailed results are presented in Table 7 and Fig 12.

**Table 7:** Impact of vehicle capacity on optimization objectives.

Vehicle capacity ($W$)	Optimal transportation risk	Optimal transportation cost	Optimal average customer satisfaction	Number of vehicles in use
1.5t	0.611	5795.78	0.547	10
2t	0.558	5128.36	0.585	8
2.5t	0.523	4653.67	0.621	7
3t	0.498	4589.23	0.635	6
3.5t	0.495	4563.85	0.636	5

As vehicle capacity increased from 1.5 to 3.5t, the optimal transportation risk showed a continuous downward trend, with an overall reduction of 19.0%. The risk reduction rate exhibited distinct phased characteristics: the largest decrease occurred when capacity rose from 1.5 to 2t, and the reduction rate slowed significantly after reaching 2.5t. This phenomenon stems primarily from two factors. First, higher vehicle capacity reduces the number of vehicles needed for transportation, thereby decreasing the total number of transport routes and the cumulative risk exposure probability. Second, larger capacity enables more flexible route planning, making it possible to avoid high-population-density areas that could not be bypassed due to load restrictions. However, when capacity exceeds 3t, the marginal effect of risk reduction diminishes—core high-risk areas have already been effectively avoided, and the remaining risk is mainly determined by the inherent characteristics of the routes.

The optimal transportation cost demonstrates a monotonically decreasing trend with increasing vehicle capacity, declining overall by 21.2%. Notably, the rate of cost reduction exhibits an unbalanced pattern similar to that of risk: it is most pronounced when capacity rises from 1.5 to 2t and diminishes significantly after exceeding 2.5t. This phenomenon is attributable to three primary factors: a reduction in the vehicle fleet directly lowers fixed costs ; fewer active routes decrease total travel distance and associated energy consumption; and a smaller fleet simplifies charging scheduling, reducing detour-induced time and cost penalties. Once capacity reaches 3t, the transportation network approaches its optimal state, leaving minimal potential for additional cost savings.

In parallel, average customer satisfaction shows continuous improvement, increasing from 0.547 to 0.636—an overall rise of 16.3%. The growth rate remains relatively stable initially before slowing considerably. This trend occurs because greater vehicle capacity enables more consolidated and efficient route planning, reducing scheduling conflicts that lead to missed time windows. Furthermore, fewer vehicles enhance operational flexibility and decrease on-site customer waiting times. As capacity surpasses 3t, customer satisfaction nears its theoretical ceiling, as most time window demands are satisfied, and further gains are constrained by physical route constraints.

**Fig. 13:**
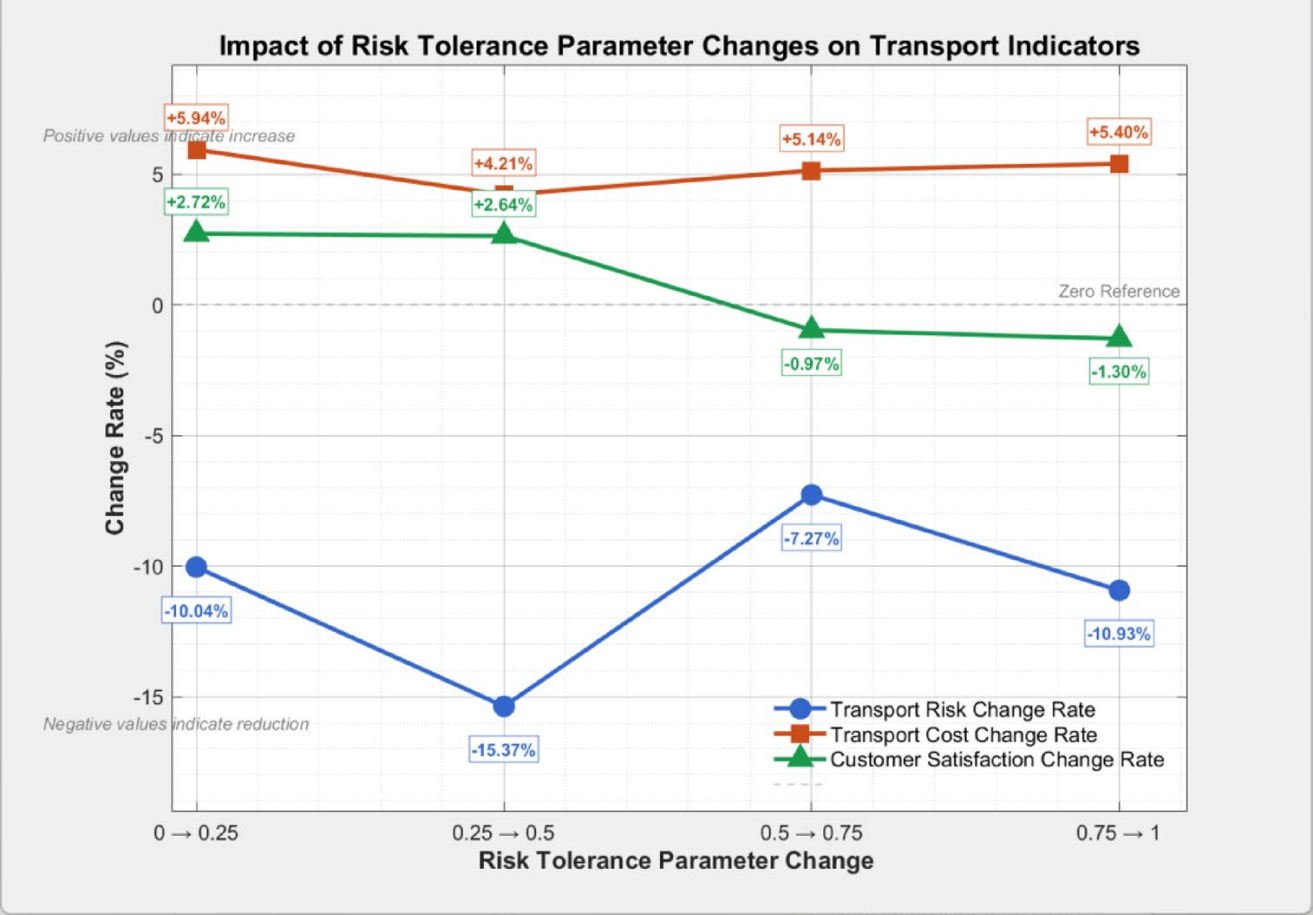
Change rates of optimization objectives across different risk-tolerance parameter intervals.

To investigate the impact of the risk tolerance parameter ($\theta_{ij}$) on the study findings, comparative experiments were conducted with $\theta_{ij}$ values set to 0, 0.25, 0.5, 0.75, and 1, while keeping all algorithm parameters consistent. The optimization objectives were used as evaluation metrics, with detailed results presented in Table 8 and Fig. 13.

**Table 8:** Optimization results under different risk-tolerance parameters.

Risk tolerance parameter ($\theta_{ij}$)	Optimal transportation risk	Optimal transportation cost	Optimal average customer satisfaction
0	0.687	4215.39	0.589
0.25	0.618	4465.87	0.605
0.5	0.523	4653.67	0.621
0.75	0.485	4892.74	0.615
1	0.432	5156.87	0.607

As $\theta_{ij}$ increases from 0 to 1, transportation risk exhibits a continuous downward trend, achieving an overall reduction of 37.1%. The risk reduction rate is most pronounced in the interval 0.25 → 0.5, indicating that a moderate level of risk aversion ($\theta_{ij} = 0.5$) yields a significant risk reduction. When $\theta_{ij} > 0.5$, the reduction rate slows, as the model gradually approaches the risk control limit under the worst-case scenario.

The total transportation cost exhibits a monotonically increasing trend with the increase in $\theta_{ij}$, registering an overall growth of 22.3%. The cost growth rate remains relatively stable across all intervals, with the most significant upturn observed in the 0 → 0.25 range. This phenomenon reflects that risk aversion incurs additional costs, including those associated with detouring high-risk areas and implementing more frequent charging scheduling.

The average customer satisfaction demonstrates a trend of first rising and then declining, peaking at $\theta_{ij} = 0.5$. Specifically, it increases by 2.7% and 2.6% in the initial stages, followed by a decrease of 0.97% and 1.3% in subsequent intervals. This pattern arises because moderate risk aversion can strike a balance between route rationality and time window adherence, whereas excessive risk aversion ($\theta_{ij} > 0.5$) results in unduly long routes and a consequent decline in satisfaction.

## Conclusion

This study centers on the multi-objective route optimization problem of hazardous materials transportation via EVs in uncertain environments. It aims to fill the research gaps in existing literature, such as inadequate consideration of uncertainties, over-reliance on single optimization objectives, and poor adaptability to the unique characteristics of EVs. A comprehensive multi-objective optimization model and an improved solution algorithm are proposed, with the following key contributions and findings:

### Research contributions

First, the study innovatively integrates uncertain factors into the risk assessment system. By quantifying the impacts of population density uncertainty (expressed as interval numbers) and dynamic cargo volume changes on transportation risks, it overcomes the limitations of traditional risk models that rely on deterministic parameters, making the risk assessment more in line with real transportation scenarios. Second, a three-objective optimization framework is constructed, simultaneously minimizing transportation risks, reducing logistics costs, and maximizing customer satisfaction. This framework balances safety, economy, and service quality, filling the gap of single or dual-objective optimization in existing EV HMT research. Third, an improved non-dominated sorting genetic algorithm (H-NSGA-II) is designed by fusing the greedy algorithm with the traditional NSGA-II. The introduction of a hybrid initialization strategy, three types of neighborhood operators, and a feasibility repair mechanism ensures the algorithm’s efficiency in obtaining high-quality Pareto solutions, especially in handling constraints such as EV battery capacity and charging needs.

### Key findings

Case simulation results show that the H-NSGA-II algorithm outperforms the standard NSGA-II in multiple dimensions: the optimal transportation risk is reduced by 14.40%, the optimal transportation cost is decreased by 12.81%, and the average customer satisfaction is increased by 13.53%. Meanwhile, the algorithm exhibits superior computational efficiency and solution diversity, with a hypervolume index improved from 0.685 to 0.752. Sensitivity analysis reveals the dynamic impacts of key parameters: as vehicle capacity increases from 1.5t to 3.5t, transportation risk and cost show a phased decreasing trend with diminishing marginal effects, while customer satisfaction continues to rise and approaches the theoretical ceiling after 3t; as the risk tolerance parameter increases from 0 to 1, transportation risk decreases by 37.1% overall, transportation cost increases by 22.3%, and customer satisfaction presents a “first rise then fall” trend peaking at $\theta_{ij} = 0.5$. Additionally, the three-dimensional conflict analysis based on correlation coefficients and MRS indicates moderate conflicts between transportation risk and cost, as well as between satisfaction and risk, and a weak positive correlation between cost and satisfaction, providing quantitative support for decision-makers to balance multiple objectives.

### Practical implications

The research results offer important decision-making support for the safe, economical, and green distribution of urban Category 9 hazardous materials. For government regulatory departments, the risk assessment method considering uncertainties can provide a basis for formulating safety standards for EV HMT; for logistics enterprises, the Pareto solution set generated by the H-NSGA-II algorithm allows flexible selection of route plans according to different priority scenarios (risk-priority, cost-priority, or satisfaction-priority). The model’s consideration of EV charging needs and load-dependent energy consumption also provides technical guidance for the application of new energy vehicles in the hazardous materials transportation field.

### Limitations and future directions

Despite its contributions, this study has certain limitations. First, the model assumes constant vehicle speed and ignores the impacts of traffic congestion, road type, and weather on transportation risks and energy consumption, which may simplify real-world complexity. Second, the risk aggregation method does not consider spatial correlations between road segments, potentially leading to conservative risk estimates. Third, the validation is based on a single test case, and the model’s adaptability to large-scale and multi-region scenarios needs further verification. Future research will focus on three aspects: integrating dynamic traffic data and multi-factor risk assessment indicators to improve model realism; adopting GIS-based gridded population data to optimize risk aggregation accuracy; and expanding comparative experiments with other multi-objective algorithms (such as MOPSO and MOEA/D) while conducting field trials with logistics partners to enhance the model’s practical application value. Additionally, exploring model compression techniques to develop a lightweight version of the algorithm will broaden its applicability in resource-constrained edge devices.

## Data Availability

The data that support the findings of this study are available from the corresponding author upon reasonable request.
